# *In vivo* correction of anaemia in β-thalassemic mice by γPNA-mediated gene editing with nanoparticle delivery

**DOI:** 10.1038/ncomms13304

**Published:** 2016-10-26

**Authors:** Raman Bahal, Nicole Ali McNeer, Elias Quijano, Yanfeng Liu, Parker Sulkowski, Audrey Turchick, Yi-Chien Lu, Dinesh C. Bhunia, Arunava Manna, Dale L. Greiner, Michael A. Brehm, Christopher J. Cheng, Francesc López-Giráldez, Adele Ricciardi, Jagadish Beloor, Diane S. Krause, Priti Kumar, Patrick G. Gallagher, Demetrios T. Braddock, W. Mark Saltzman, Danith H. Ly, Peter M. Glazer

**Affiliations:** 1 Department of Therapeutic Radiology, Yale University, New Haven, Connecticut 06520, USA; 2 Department of Biomedical Engineering, Yale University, New Haven, Connecticut 06511, USA; 3 Department of Genetics, Yale University, New Haven, Connecticut 06520, USA; 4 Department of Laboratory Medicine, Yale University, New Haven, Connecticut 06520, USA; 5 Department of Chemistry and Center for Nucleic Acids Science and Technology (CNAST), Carnegie Mellon University, Pittsburgh, Pennsylvania 15213, USA; 6 Program in Molecular Medicine, University of Massachusetts Medical School, Worcester, Massachusetts 01605, USA; 7 Yale Center for Genome Analysis (YCGA), Yale University, Orange, Connecticut 06477, USA; 8 Department of Internal Medicine, Section of Infectious Disease, Yale University, New Haven, Connecticut 06520, USA; 9 Department of Pediatrics, Yale University, New Haven, Connecticut 06520, USA; 10 Department of Pathology, Yale University, New Haven, Connecticut 06520, USA

## Abstract

The blood disorder, β-thalassaemia, is considered an attractive target for gene correction. Site-specific triplex formation has been shown to induce DNA repair and thereby catalyse genome editing. Here we report that triplex-forming peptide nucleic acids (PNAs) substituted at the γ position plus stimulation of the stem cell factor (SCF)/c-Kit pathway yielded high levels of gene editing in haematopoietic stem cells (HSCs) in a mouse model of human β-thalassaemia. Injection of thalassemic mice with SCF plus nanoparticles containing γPNAs and donor DNAs ameliorated the disease phenotype, with sustained elevation of blood haemoglobin levels into the normal range, reduced reticulocytosis, reversal of splenomegaly and up to 7% β-globin gene correction in HSCs, with extremely low off-target effects. The combination of nanoparticle delivery, next generation γPNAs and SCF treatment may offer a minimally invasive treatment for genetic disorders of the blood that can be achieved safely and simply by intravenous administration.

## Abstract (web summary)

Gene editing approaches are widely used for correcting mutations, but their application is largely limited to cells and not living animals. Here the authors show that *in vivo* γPNA-mediated editing of a β-globin mutation is promoted by SCF and leads to sustained normalization of blood haemoglobin levels β-thalassemic mice.

## Graphical abstract

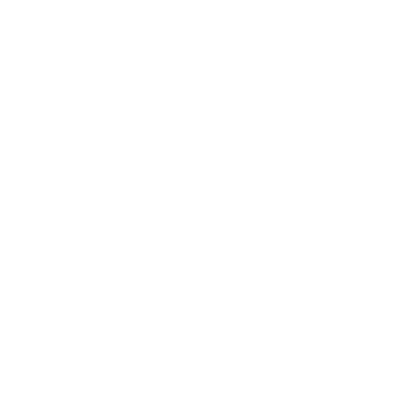

Gene editing in haematopoietic stem cells (HSCs) may provide a strategy for treatment of inherited disorders such as sickle cell disease and β-thalassaemia. Methods for gene editing include targeted nucleases such as zinc-finger nucleases^1^ and CRISPR/Cas9 (ref. 2), short fragment homologous recombination^3^ and triplex-forming oligonucleotides^4^. Recent excitement has focused on CRISPR/Cas9 technology because of facile reagent design^5^. However, like zinc-finger nucleases, the CRISPR approach introduces an active nuclease into cells, which can lead to off-target cleavage in the genome^6^. As an alternative, we have pursued triplex-forming peptide nucleic acids (PNAs) designed to bind site-specifically to genomic DNA via strand invasion and formation of PNA/DNA/PNA triplexes (via both Watson–Crick and Hoogsteen binding) with a displaced DNA strand^7^,^8^,^9^. PNAs consist of a charge-neutral peptide-like backbone and nucleobases enabling hybridization with DNA with high affinity. PNA/DNA/PNA triplexes recruit the cell's endogenous DNA repair systems to initiate site-specific modification of the genome when single-stranded ‘donor DNAs' are co-delivered as templates containing the desired sequence modification^10^.

Our prior work has suggested that PNA-induced genome modification is mediated by the nucleotide excision repair and homology-dependent repair (HDR) pathways^10^,^11^. Both nucleotide excision repair and HDR are high fidelity pathways, and the PNAs lack any intrinsic nuclease activity; together these features may account for the very low frequencies of off-target genotoxicity seen with PNA-mediated gene editing compared with nuclease based approaches^12^,^13^,^14^. We have also recently shown that tail-clamp PNAs (tcPNAs) with an extended Watson–Crick binding domain can enhance gene editing in human haematopoietic cells with increased efficiency and specificity^13^ and that polymer nanoparticles (NPs) can effectively deliver these molecules into human HSCs both *ex vivo* and *in vivo* in a humanized mouse model^12^,^15^.

Here, we use next generation triplex-forming γPNAs containing a polyethylene glycol substitution at the gamma position for enhanced DNA binding^16^. Delivered via polymer NPs, the γPNAs mediate increased gene editing in HSCs both *ex vivo* and *in vivo* in two different mouse models, one carrying a β-globin/green fluorescent protein (GFP) fusion transgene and the other carrying the human β-globin gene replacing the mouse β-globin genes and containing a β-thalassaemia-associated splicing mutation at IVS2-654 (refs 17, 18). We find that treatment with stem cell factor (SCF), the c-Kit ligand, further enhances PNA-mediated gene editing, an effect associated with increased DNA repair. Treatment of thalassemic mice^18^ with NPs containing γPNAs and donor DNAs, plus SCF, produced up to 7% gene editing in HSCs, with elevation of blood haemoglobin levels for 140 days post-treatment, reduction in reticulocyte counts and reversal of splenomegaly. γPNA treatment also yielded gene editing in human CD34+ HSCs *ex vivo* at a frequency of 5% with a single treatment, with very low off-target effects. These results indicate that chemical advances in PNA design and *in vivo* delivery via polymer NPs can mediate clinically relevant levels of gene editing. They also identify SCF treatment as a potential pharmacologic strategy to increase gene editing that may be applicable not only to triplex-forming PNAs but also to approaches such as CRISPR/Cas9.

## Results

### Triplex-forming PNA design for gene editing

**Figure 1:**
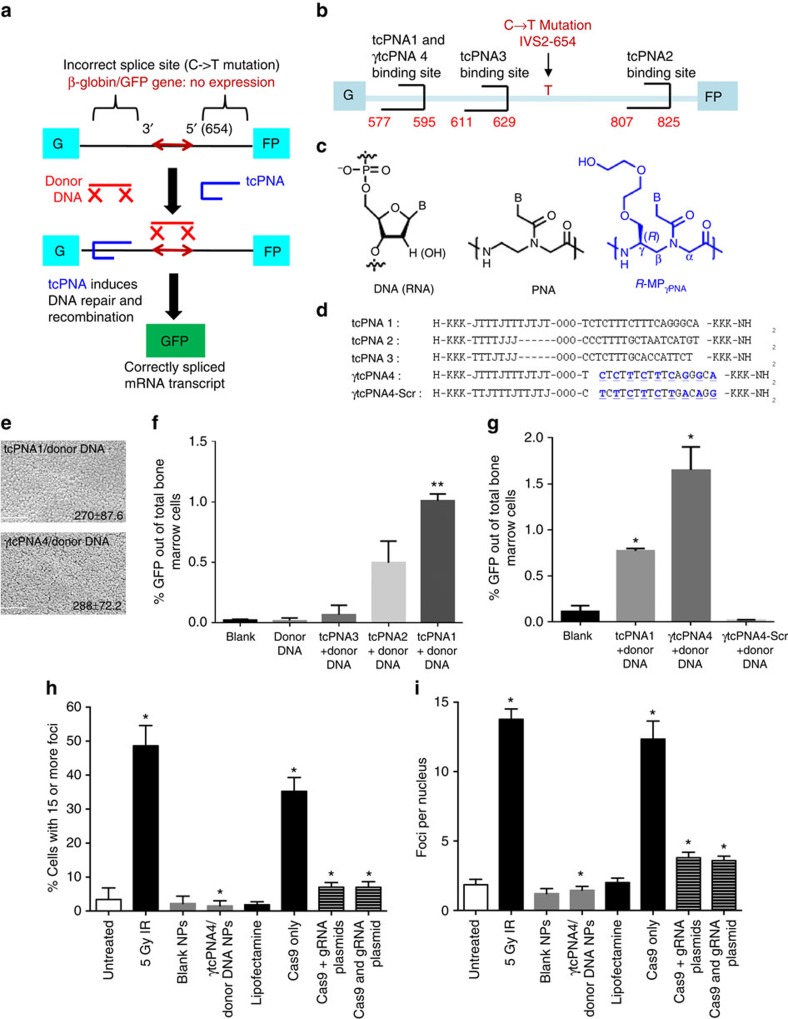
Gene editing using γPNAs in mouse bone marrow. (**a**) Strategy for targeted correction of a β-globin gene IVS2-654 (C->T) mutation in β-globin/GFP transgenic mice using triplex-forming tail-clamp PNAs and donor DNAs. (**b**) tcPNA and γtcPNAs designed to bind to homopurine regions within intron 2 of the human β-globin gene near IVS2-654 (C->T). (**c**) DNA, unmodified PNA and ^MP^γPNA. (**d**) tcPNAs and γtcPNAs to bind to the positions indicated in B. γtcPNA4-Scr is a scrambled version of γtcPNA4. Bold/underline indicates γPNA residues. K indicates lysine; J, pseudoisocytosine (for **c**) for pH-independent triplex formation. O, 8-amino-2,6,10-trioxaoctanoic acid linkers connecting the Hoogsteen and Watson–Crick domains of the tcPNAs. (**e**) Scanning electron microscope images of nanoparticles. Scale bar, 2.0 μm. (**f**) Gene correction of the IVS2-654 (C->T) mutation within the β−globin/GFP fusion gene in mouse BM cells treated *ex vivo* with NPs containing the indicated tcPNAs and donor DNA. %GFP+ was determined by flow cytometry and indicates successful gene editing. Data are mean±s.e., *n*=3; statistical analysis by Student's *t*-test, **P<0.005. (**g**) %GFP+ cells in mouse BM after *ex vivo* treatment with NPs containing tcPNA1, γtcPNA4 or γtcPNA4-Scr, plus donor DNAs. Data are mean±s.e.m., *n*=3; analysis by Student's *t*-test, *P<0.05. (**h**,**i**) Quantification of γH2AX foci by immune fluorescence microscopy, indicative of DNA DSBs in primary fibroblasts (from the β-globin/GFP transgenic mice) either untreated or treated with 5 Gy of IR, blank NPs, NPs containing γtcPNA4 and donor DNA, lipofectamine alone, lipofectamine transfection of a Cas9 expression vector, lipofectamine transfection of a Cas9 vector and a separate guide RNA expression vector (targeting the same site in β-globin sequences as γtcPNA4; Cas9+gRNA), or transfection of a vector containing both Cas9 and guide RNA (Cas9 and gRNA). Quantification as per cent of cells with 15 or more foci (**h**) or average number of foci per cell (**i**). 100 cells were counted per condition, data are mean±s.e.m, *n*=3; analysis by Student's *t*-test, *P<0.05.

To quantitatively assay for gene editing, we used a mouse model with a β-globin/GFP fusion transgene consisting of human β-globin intron 2 carrying a thalassaemia-associated IVS2-654 (C→T) mutation embedded within the GFP coding sequence, resulting in incorrect splicing of β-globin/GFP mRNA and lack of GFP expression^19^. PNA-mediated triplex-formation induces recombination of the genomic site with a 60-nucleotide sense donor DNA homologous to the β-globin intron 2 sequence except for providing a wild-type nucleotide at the IVS2-654 position. Correction of the splice-site mutation yields expression of functional GFP (Fig. 1a)^12^,^15^, providing a phenotypic read-out of editing quantifiable by flow cytometry.

We designed a series of tcPNAs to bind to polypurine stretches in the β-globin intron near the IVS2-654 mutation (Fig. 1b). One of the tcPNAs and a scrambled sequence control were synthesized to contain partial substitution with a mini-polyethylene-glycol group at the γ position (^MP^γPNA; Fig. 1c,d) within their Watson–Crick binding domains. We made the substitutions in the Watson–Crick domains because in prior work γPNAs have been shown to enhance strand invasion and DNA binding in the Watson–Crick binding mode due to helical pre-organization enforced by the modification^16^. We hypothesized that this would enhance the binding of the tcPNAs because strand invasion and Watson–Crick PNA/DNA duplex formation is an important component in the formation of PNA/DNA/PNA triplexes. Partial substitution was performed because it is sufficient to improve binding affinity and to confer helical pre-organization^20^. γtcPNA4 matches the sequence of tcPNA1 except that it contains γ units at alternating positions in the Watson–Crick domain (Fig. 1d). Scrambled γtcPNA (γtcPNA4-Scr) has the same base composition as γtcPNA4 but a scrambled sequence. All tcPNA oligomers were synthesized with 3 lysines at both termini to improve solubility and increase binding affinity (Fig. 1d). Purification and characterization of the synthesized PNAs were performed by high-performance liquid chromatography analyses and matrix-assisted laser desorption/ionization time-of-flight, respectively (Supplementary Fig. 1 and Supplementary Table 1). Gel shift assays to assess the binding of the tcPNAs to DNAs containing the respective target sequences showed that all bound specifically to their target sites (Supplementary Fig. 2A). No binding was seen in the case of the scrambled sequence γtcPNA4-Scr.

In prior work, we showed that poly(lactic-co-glycolic acid) (PLGA) NPs can effectively deliver PNA/donor DNA combinations into primary human and mouse haematopoietic cells with essentially no toxicity^12^,^14^,^21^. Here, tcPNAs and donor DNAs, at a molar ratio of 2:1, were incorporated into poly(lactic-co-glycolic acid) (PLGA) NPs. The NP formulations were evaluated by scanning electron microscopy and dynamic light scattering. All the NPs exhibited sizes within the expected range (Fig. 1e and Supplementary Fig. 2B,C) and showed uniform charge distribution (Supplementary Fig. 2D). Nucleic acid release profiles showed that γ modifications did not impair release from NPs (Supplementary Fig. 2E).

### *Ex vivo* gene editing in bone marrow cells

Bone marrow (BM) cells harvested from β-globin/GFP mice were treated *ex vivo* with PLGA NPs containing tcPNA1/donor DNA, tcPNA2/donor DNA and tcPNA3/donor DNA. After 48 h, the percentage of GFP+ (corrected) cells was quantified via flow cytometry (Fig. 1f). The higher gene editing activity of tcPNA1 is likely due to its longer Hoogsteen binding domain, an effect that we have previously observed^13^. NPs containing the γ-substituted tcPNA (γtcPNA4) and donor DNA yielded significantly higher gene modification (1.62%; Fig. 1g and Supplementary Fig. 3A), showing that the ^MP^γ substitutions confers increased biological activity that correlates with their improved binding properties. NPs with the γtcPNA4–Scr produced no modification (Fig. 1g).

BM cells treated with either blank NPs or NPs containing γtcPNA4/donor DNA were plated in methylcellulose medium supplemented with cytokines for growth of granulocyte/macrophage colonies (colony-forming unit (CFU)-G, CFU-M and CFU-GM) or combined colonies (CFU-GEMM, granulocyte, erythroid, monocyte/macrophage and megakaryocyte). The two sets of treated cells formed myeloid and erythroid colonies at similar frequencies (Supplementary Fig. 3B,C), suggesting that treatment with γtcPNA4 and donor DNA does not impair the ability of the progenitor cells to proliferate and differentiate. Sequencing analysis of genomic DNA from selected GFP-positive methylcellulose colonies confirmed the presence of the targeted gene modification in the β-globin/GFP transgene at the IVS2-654 base pair (Supplementary Fig. 3D). Also, there was no induction of the inflammatory cytokines in the treated BM cells (Supplementary Fig. 3E), consistent with prior work with NPs containing standard PNAs^12^,^14^,^21^.

In assays for genotoxicity, there was no detectable increase in DNA double-strand breaks (DSBs) in the BM cells treated with γtcPNA4/donor DNA-containing NPs compared with blank NPs based on a single-cell gel electrophoresis assay (Comet assay; Supplementary Fig. 4A). We also assayed for DSBs by measuring the production of γH2AX foci. γH2AX foci are detected in nuclei by immune fluorescence and document a chromatin modification that occurs upon DSB formation via DNA damage response signalling^22^. Treatment of the BM cells with NPs containing γtcPNA4/donor DNA did not produce any γH2AX foci above the low background that is seen in untreated cells (assayed by flow cytometry as shown in Supplementary Fig. 4B). Treatment of the cells with 5 Gy of ionizing radiation (IR) was included as a positive control for induction of DSBs. We also used primary fibroblasts from the mice for this assay, since adherent cells facilitate more robust quantification of foci by immune fluorescence microscopy. Treatment of the fibroblasts with NPs containing γtcPNA4/donor DNA again did not produce γH2AX foci above the low background in untreated or blank NP treated cells (quantified in Fig. 1h as per cent of cells with more than 15 foci and in Fig. 1i as average number of foci per nucleus, with corresponding immune fluorescence images of foci shown in Supplementary Fig. 5). For comparison, transfection of a vector expressing the Cas9 nuclease yielded an increase in γH2AX foci (Fig. 1h,i). Co-expression of a guide RNA designed to bind to the same sequence in the β-globin gene as γtcPNA4 (either via the same plasmid or via a plasmid separate from the Cas9 vector) reduced the number of induced DSBs, but to a level that was still above background (Fig. 1h,i and Supplementary Fig. 5).

### Elevated gene editing by γtcPNAs in CD117+ cells

**Figure 2:**
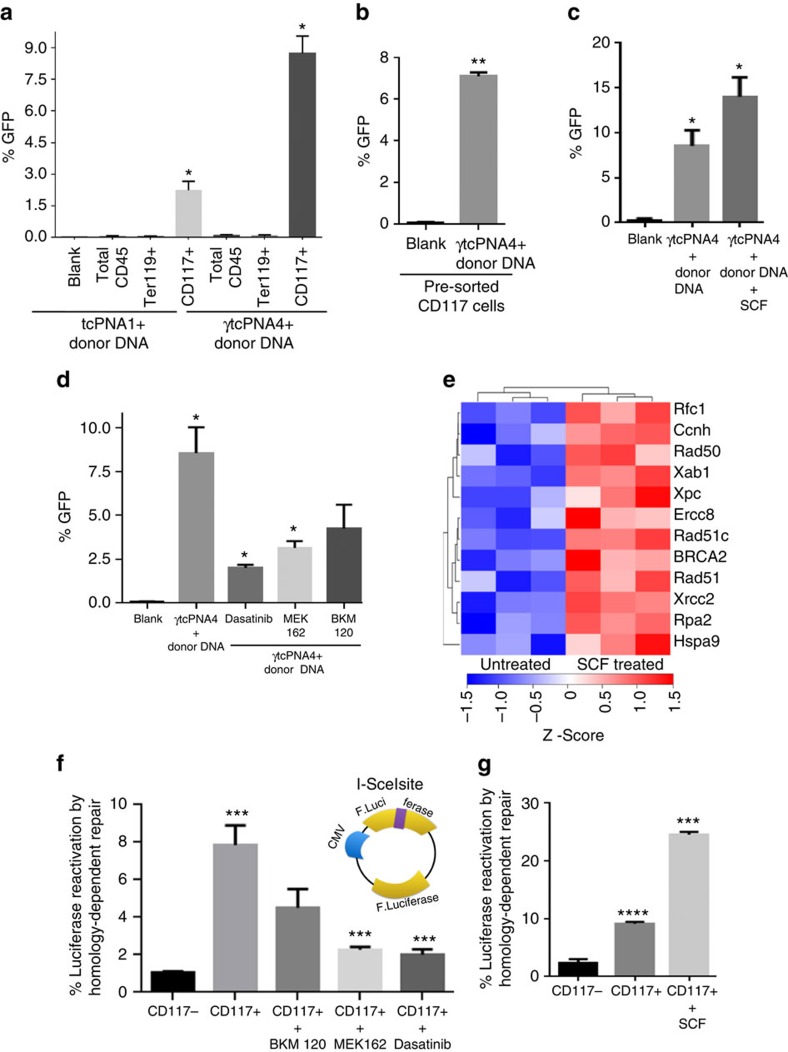
The SCF/c-Kit+ pathway promotes gene editing and DNA repair. (**a**) %GFP expression in treated mouse BM cells expressing the indicated cell surface markers. Total BM was treated with NPs containing either tcPNA1/donor DNA or γtcPNA4/donor DNA, cells were stained using antibodies specific for the indicated markers and assayed by flow cytometry for marker and GFP expression. Data are mean±s.e.m, *n*=3; statistical analysis by Student's *t*-test, *P<0.05. (**b**) %GFP expression in pre-sorted CD117 (c-Kit+) cells treated with either NPs carrying γtcPNAs and donor DNAs or blank NPs. Data are mean±s.e.m., *n*=3; statistical analysis by Student's *t*-test, **P<0.005. (**c**) %GFP expression in pre-sorted CD117+ cells treated with NPs containing γtcPNA4/donor DNA with or without prior treatment with the c-Kit ligand, SCF. Data are mean±s.e.m., *n*=3; statistical analysis by Student's *t*-test, *P<0.05. (**d**) %GFP expression in pre-sorted CD117+ cells treated with NPs containing γtcPNA4/donor DNA in the presence or absence of c-Kit pathway kinase inhibitors: dasatinib (inhibits c-Kit), MEK162 (inhibits MEK, MEK) and BKM120 (inhibits phosphatidylinositol-3-kinase). Data are mean±s.e.m, *n*=3; statistical analysis by Student's *t*-test, *P<0.05. (**e**) Heat map showing upregulated genes involved in DNA repair in CD117+ cells with or without SCF treatment; rows are clustered by Euclidean distance measure. (**f**) Reporter gene assay for HDR activity in CD117 cells in the presence or absence of c-Kit pathway kinase inhibitors: dasatinib, MEK162 and BKM120 (as above). Inset shows diagram of the luciferase reporter assay for repair of a nuclease-indcued DSB by HDR. Luciferase expression occurs only after homologous recombination and is scored as % reactivation of the DSB-damaged plasmid, normalized to a transfection control. Data are mean±s.e.m., *n*=3; statistical analysis by Student's *t*-test, ***P<0.005. (**g**) HDR assay in CD117+ cells with or without the addition of SCF. Data are shown as mean±s.e.m., *n*=3; statistical analysis by Student's *t*-test, ***P<0.005 and ****P<0.0005.

Previous work suggested that there might be increased PNA-mediated editing in colony-forming progenitors^12^. To test this, we treated whole BM cells with either blank NPs, NPs containing tcPNA1/donor DNA, or NPs containing γtcPNA4/donor DNA. Two days later, flow cytometry was performed to assess the frequency of GFP+ cells within selected sub-populations. CD117+ cells showed elevated gene editing compared with the total CD45+ cell population (Fig. 2a), with 8.6% in CD117+ cells after a single treatment with γtcPNA4/donor DNA NPs. The less potent tcPNA1/donor DNA NPs still yielded an elevated correction frequency of 2.1% in CD117+ cells compared with the total BM (although fourfold lower than γtcPNA4). Next, we sorted for CD117+ cells before treatment with the NPs (Fig. 2b). An elevated percentage of modification (7.2%) was again seen after a single treatment (Fig. 2b).

### The c-Kit pathway mediates increased gene editing

CD117 (also known as mast/stem cell growth factor receptor or proto-oncogene c-Kit protein) is a receptor tyrosine kinase expressed on the surface of haematopoietic stem and progenitor cells and other cell types. SCF, the ligand for c-Kit, causes dimerization of the receptor, activating its kinase activity to trigger signalling pathways that impact survival, proliferation and differentiation.

We asked whether c-Kit-dependent signalling is required for elevated gene correction or whether CD117 simply serves as a marker for the increased susceptibility. First, we tested for gene editing in pre-sorted CD117+ cells treated with or without the c-Kit ligand, SCF, and we observed a significant increase in γtcPNA4/donor DNA-mediated gene editing (up to almost 15%) in the SCF-treated cells (Fig. 2c and Supplementary Fig. 6). We next assayed for γtcPNA4/donor DNA NP-mediated gene editing in pre-sorted CD117+ cells in the presence or absence of kinase inhibitors (Fig. 2d). Dasatinib, which inhibits the c-Kit kinase in addition to the BCR/Abl and Src kinases, reduced the gene editing from 7 to 2.0%. Inhibitors of factors downstream of c-Kit, including mitogen/extracellular signal-regulated kinase (MEK; Binimetinib; MEK162) and phosphatidylinositol-3-kinase (BKM120), also decreased gene editing in CD117+ cells to 2.6 and 4.1%, respectively (Fig. 2d).

We also tested tcPNA1 (no gamma substitutions) for gene editing in pre-sorted CD117 cells treated or not with SCF. We found that SCF boosted tcPNA1/donor DNA gene editing up to 5% versus about 1.5% without SCF (Supplementary Fig. 7). While substantial, this was threefold lower than the almost 15% frequency seen with the γtcPNA4/donor DNA NPs in conjunction with SCF treatment of the pre-sorted CD117 cells (Fig. 2c). Overall, these results indicate that the SCF/c-Kit signalling can enhance gene editing and identify SCF as a potential agent to stimulate PNA-mediated gene modification. In addition, the results further show that γtcPNAs provide increased gene editing potential as compared with regular tcPNAs.

### Increased DNA repair gene expression upon activation of c-Kit+

To explain the increased gene editing in CD117 cells, we tested whether CD117 might be associated with differential uptake of the NPs. However, there were no differences in uptake across several BM cell sub-populations (Supplementary Fig. 8). Next, we examined gene expression patterns in the c-Kit+ cells to test the hypothesis that there might be increased DNA repair gene expression. RNA from sorted CD117+ and CD117− cells from the β-globin/GFP mice was analysed using Illumina arrays (Supplementary Fig. 9A–C). We found that numerous DNA repair genes, including *BRCA1, BRCA2, Rad51, ERCC2, XRCC2* and *XRCC3*, showed higher levels of expression in CD117+ cells (Supplementary Figs 10 and 11a)., The elevated expression two genes likely to play a role in PNA-induced recombination, *BRCA2* and *Rad51*, was confirmed in CD117+ cells by quantitative PCR with reverse transcription (Supplementary Fig. 11B,C) and by western blot (Supplementary Fig. 11D). We went on to test whether SCF treatment could further increase DNA repair gene expression. Gene expression profiling showed additional up-regulation of numerous DNA repair genes by SCF treatment (Fig. 2e and Supplementary Fig. 12A,B).

Because SCF, as a stimulatory cyctokine, would be expected to impact the cell cycle in c-Kit+ cells and because HDR gene expression is known to be elevated in S-phase, we also measured the effect of SCF on cell cycle phase distribution in the pre-sorted CD117 cells. We found that there was a 9% increase in the per cent of cells in S-phase at 48 h after SCF treatment as compared with no SCF treatment (Supplementary Fig. 13). Hence, some component of the SCF effect on DNA repair genes could be an indirect effect of cell cycle changes.

### The c-Kit pathway induces functionally elevated DNA repair

To test whether the above increases in DNA repair gene expression could be correlated with functional differences in DNA repair, we used a luciferase-based assay to quantify repair of DNA DSBs by HDR. In this assay, repair of a DSB in a reporter plasmid via intramolecular homologous recombination creates (‘reactivates') a functional luciferase gene (Fig. 2f), and so the assay provides a measure of HDR capacity (validation of the assay is shown in Supplementary Fig. 14). The results show increased HDR in CD117+ compared with CD117− cells (Fig. 2f). HDR in CD117+ cells was diminished by the kinase inhibitors MEK162, BKM120 and dasatnib (Fig. 2g); conversely, it was boosted by SCF treatment (Fig. 2g). These results indicate that c-Kit signalling increases HDR.

### *In vivo* gene editing by PNA/DNA NPs given intravenously

**Figure 3:**
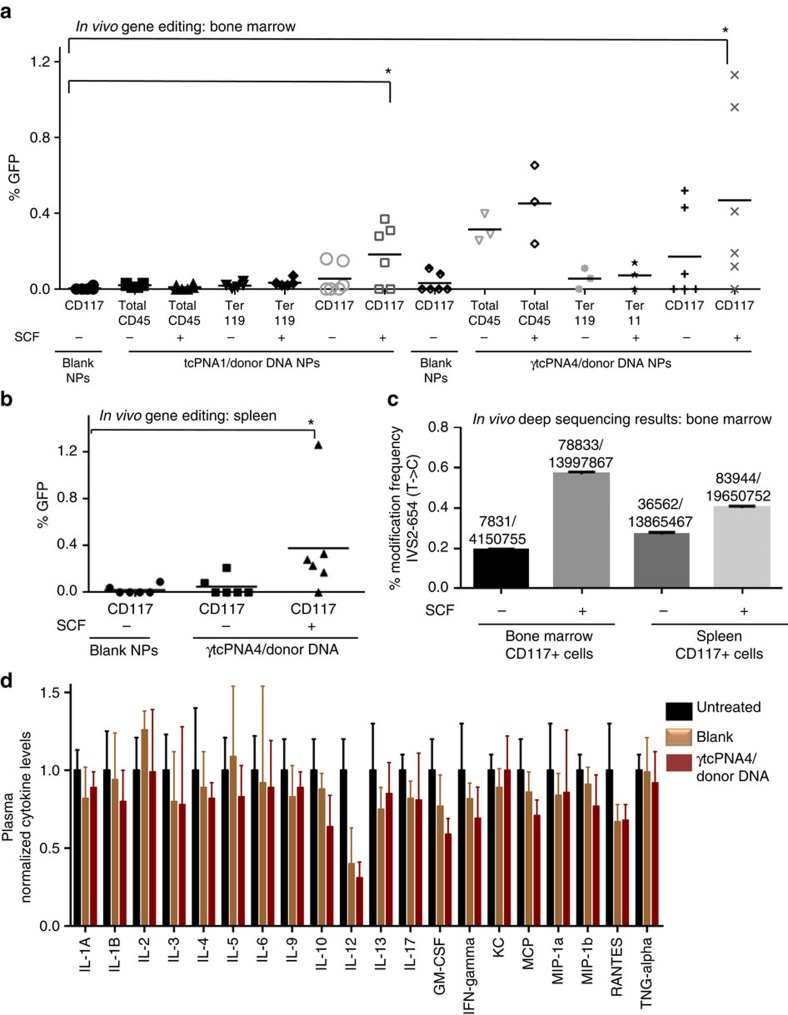
SCF treatment of mice enhances *in vivo* gene editing by γtcPNAs and donor DNAs. (**a**,**b**) β-globin/GFP transgenic mice (*n*=6 mice per group) were injected or not (as indicated) with 220 μg kg^−1^ of SCF i.p. followed by a single treatment of 4 mg of NPs injected intravenously. Each group received either blank NPs, NPs containing tcPNA1 and donor DNA, or NPs containing γtcPNA4 and donor DNA, with or without SCF. Two days later, BM and spleen cells were harvested for analysis by flow cytometry and deep sequencing. Frequencies of gene editing in haematopoietic cell sub-populations identified by the indicated cell surface markers from BM (**a**) and spleen (**b**) of mice treated with the indicated NPs and with or without pre-treatment with SCF. Each data point represents analysis of cells from a single mouse. Horizontal bars indicate mean, statistical analysis by Student's unpaired *t*-test, *P<0.05. (**c**) Deep-sequencing analysis to quantify the frequency of targeted gene editing *in vivo* in CD117+ cells from BM and spleen of β-globin/GFP mice treated as above. Error bars indicate standard error of proportions. (**d**) Analysis of cytokine levels, as indicated, in blood of either untreated, blank NP treated, or γtcPNA4 and donor DNA NP treated mice at 48 h post treatment. Data are shown as mean±s.e.m., *n*=3.

We next tested the potential for *in vivo* gene editing in the β-globin/GFP transgenic mice by simple intravenous injection of NPs containing tcPNA1/donor DNA or γtcPNA4/donor DNA, and we further asked whether the editing could be enhanced by SCF treatment. Mice were treated with a single dose of 4 mg NPs, and 2 days later we analysed gene editing in BM and spleen. Some mice also received murine SCF (220 μg kg^−1^) given by intraperitoneal injection 3 h before the NP injection (Fig. 3a,b, with experimental scheme shown in Supplementary Fig. 15A). *In vivo* gene editing was scored by GFP expression in marker-sorted cell populations from BM and spleen (Fig. 3a,b). We observed an average of ∼0.2% gene editing in the CD117 BM cells from tcPNA1/donor DNA and SCF-treated mice (Fig. 3a). Two to threefold higher levels of gene editing were seen in CD117+ cells from BM of the γtcPNA4/donor DNA and SCF-treated mice, with frequencies in the range of 1% in several mice, and average frequencies in the 0.5% range. Similar frequencies were seen in spleen (Fig. 3b).

We confirmed these results by performing deep sequencing analysis on DNA from CD117+ cells isolated from BM and spleen of treated mice (Fig. 3c and Supplementary Fig. 15A), revealing gene editing frequencies in the range of 0.2% in the BM of mice treated with γtcPNA4/donor DNA NPs without SCF and 0.6% in mice receiving SCF along with the γtcPNA4/donor DNA NPs (in a single treatment in each case), consistent with the frequencies of gene correction quantified by GFP expression. Deep sequencing was also used to assess off-target effects in the BM cells of the mice that were treated with SCF and γtcPNA4 and donor DNA NPs (Table 1). By BLAST analysis, we identified seven off-target sites with partial homology to the target site of γtcPNA4 in β-globin intron 2. Extremely low frequencies of off-target effects were found in the γtcPNA4/donor DNA treated mice, with six sites showing no detectable sequence changes out of millions of reads and two sites showing modification frequencies of only 0.0074 and 0.00018% compared with 0.56% at the targeted β-globin site (Table 1). The overall off-target modification frequency at all seven sites combined was 0.00034%, 1,647-fold lower than the frequency of the targeted gene editing.

We also performed cytokine array analyses on plasma derived from mice 48 h after treatment with γtcPNA4/donor DNA NPs . There were no detectable increases in levels of any of the cytokines measured compared with untreated mice (Fig. 3d), with lipopolysaccharide treatment as a positive control showing significantly higher levels of multiple cytokines that were off the scale of the graph (Supplementary Fig. 16). In a separate experiment, mice were treated with γtcPNA4/donor DNA NPs on day 1 and again 3 months later, followed by cytokine analysis of peripheral blood, again showing minimal immune or inflammatory response (Supplementary Fig. 17A,B).

### *In vivo* correction of a β-thalassaemia mutation in mice

We next tested for correction of a human β-thalassaemia mutation in a mouse disease model, using a transgenic mouse line in which the two (*cis*) murine adult beta globin genes were replaced with a single copy of the human β-globin gene with the same thalassaemia-associated IVS2-654 mutation as above^18^. Homozygous mice do not survive, and heterozygotes have a moderate form of β-thalassaemia, with haemolytic anaemia, microcytosis and other erythrocyte morphologies reflecting reduced amounts of mouse β-globin and no human β-globin^17^,^18^, consistent with β-thalassaemia. Treatment groups included (1) blank NPs; (2) SCF alone (no NPs); (3) SCF plus γtcPNA4/donor DNA NPs; and (4) SCF plus γtcPNA4-Scr/donor DNA (experimental scheme is shown in Supplementary Fig. 15B). We conducted two otherwise identical replicate experiments except that, in one, the complete blood count (CBC) analyses were continued for 75 days after the last treatment, and in the other for 140 days. In both cases, each treatment group consisted of six mice, and each mouse received four treatments at 2 day intervals at the beginning of the experiment as indicated in Supplementary Fig. 15B. Some animals were maintained long-term for serial CBC analyses; others were killed at intermediate time points for analysis of spleen size and architecture and for deep sequencing of BM-derived cells.

**Figure 4:**
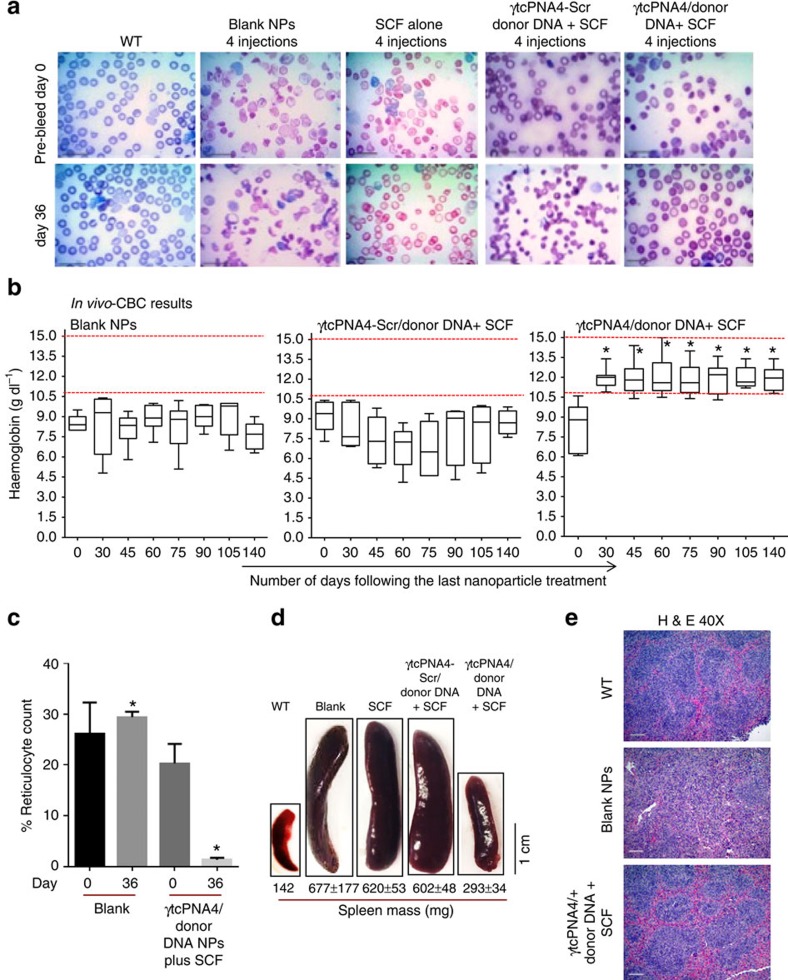
Correction of anaemia in thalassemic mice by NPs containing γtcPNA and donor DNA. (**a**) Blood smears from wild-type and thalassemic mice obtained pre-treatment or 36 days after *in vivo* treatment with blank NPs, SCF alone, SCF plus scrambled γtcPNA4-Scr/donor DNA NPs or SCF plus γtcPNA4/donor DNA NPs. NPs were given i.v.; SCF i.p. The untreated group (and control animals) exhibit extreme poikilocytosis as well as numerous target cells, cabot rings, anisochromasia and ovalocytosis, all changes characteristic of β-thalassemia. Treatment with γtcPNA4/donor DNA and SCF ameliorates the poikilocytosis and yields a reduction in anisocytosis, ovalocytosis and target cells suggestive of reduced alpha-globin precipitation in the RBCs. Scale bar, 1.0 μm. (**b**) Blood haemoglobin levels of thalassemic mice treated with blank NPs, SCF plus scrambled γtcPNA4-Scr/donor DNA NPs, or with SCF plus γtcPNA4/donor DNA NPs performed at the indicated number of days after treatment, up to 140 days. Data are presented as box and whisker plots showing the median and quartile range within each group over time (*n*=6 per group). Only the SCF plus γtcPNA4/donor DNA-treated mice achieved and maintained haemoglobin levels within the normal range during the duration of the experiment, reflecting the increased haemoglobin stability conferred by the gene editing. Horizontal bars within the boxes indicate mean; statistical analysis by Student's unpaired *t*-test, *P<0.05. (**c**) Reticulocyte counts (% of total RBCs) calculated in blood smears from thalassemic mice treated with either blank NPs or with NPs containing and γtcPNA4/donor DNA plus SCF on days 0 and 36 post treatment. Data are mean±s.e.m., *n*=3; statistical analysis by Student's *t*-test, *P<0.05. (**d**) Images of spleens from wild-type mice or thalassemic mice treated with blank NPs, SCF alone, SCF plus scrambled γtcPNA4-Scr/donor DNA NPs or SCF plus γtcPNA4/donor DNA NPs at 36 days after the last treatment. Average spleen weights (with standard errors, *n*=3; except for wild-type, where *n*=1) are listed below the images. Splenomegaly is corrected only by SCF plus γtcPNA4/donor DNA treatment. Scale bar, 1.0 cm. (**e**) Histopathologic analysis of spleen sections from wild-type mice and from thalassemic mice obtained 36 days after treatment with blank NPs or SCF plus γtcPNA4/donor DNA NPs. Haematoxylin and eosin stain (H&E), × 40 magnification. Scale bar, 10.0 μm. Additional images of spleens from other treatment groups and with additional stains are presented in Supplementary Figs 17 and 18.

Blood smears at day 0 (before treatment) and at day 36 after the last treatment (Fig. 4a) showed marked improvement in RBC morphology on day 36 in the γtcPNA4/donor DNA treated mice but not in the mice treated with either blank NPs, SCF alone or SCF plus γtcPNA4-Scr/donor DNA. CBC analyses performed on blood samples taken at 30, 45, 60, 75, 90 and 140 days post-treatment from mice in each group showed persistent correction of the anaemia based on blood haemoglobin levels in the mice treated with SCF plus the γtcPNA4/donor DNA NPs (Fig. 4b), with elevation of the blood haemoglobin levels into the normal range. The anaemia was not improved in any of the controls. We also observed reduced reticulocyte counts in mice treated with SCF plus the γtcPNA4/donor DNA NPs but not in the mice treated with blank NPs (Fig. 4c). In addition, the γtcPNA4/donor DNA treated mice also showed reduced splenomegaly at 36 days post-treatment (Fig. 4d).

Consistent with reduced splenomegaly, histologic examination of spleens from mice on days 36 and 75 showed substantially improved splenic architecture specifically in the γtcPNA4/donor DNA treated mice (Fig. 4e and Supplementary Figs 18 and 19). The regular splenic pattern of white pulp (lymphoid follicles) surrounded by rims of red pulp is disrupted in the β-thalassemic animals due to extramedullary haematopoiesis, causing an expansion in red pulp (causing the splenomegaly) and disruption of the white pulp. The CD61 and Ecad immunohistochemical stains (Supplementary Fig. 18) highlight the increased cellularity characteristic of extramedullary haematopoiesis and demonstrate that the expanded red pulp includes elevated megakaryocytes and erythroid precursors, respectively. This increased cellularity is substantially ameliorated in the γtcPNA4/donor DNA treated mice (Supplementary Fig. 18).

**Figure 5:**
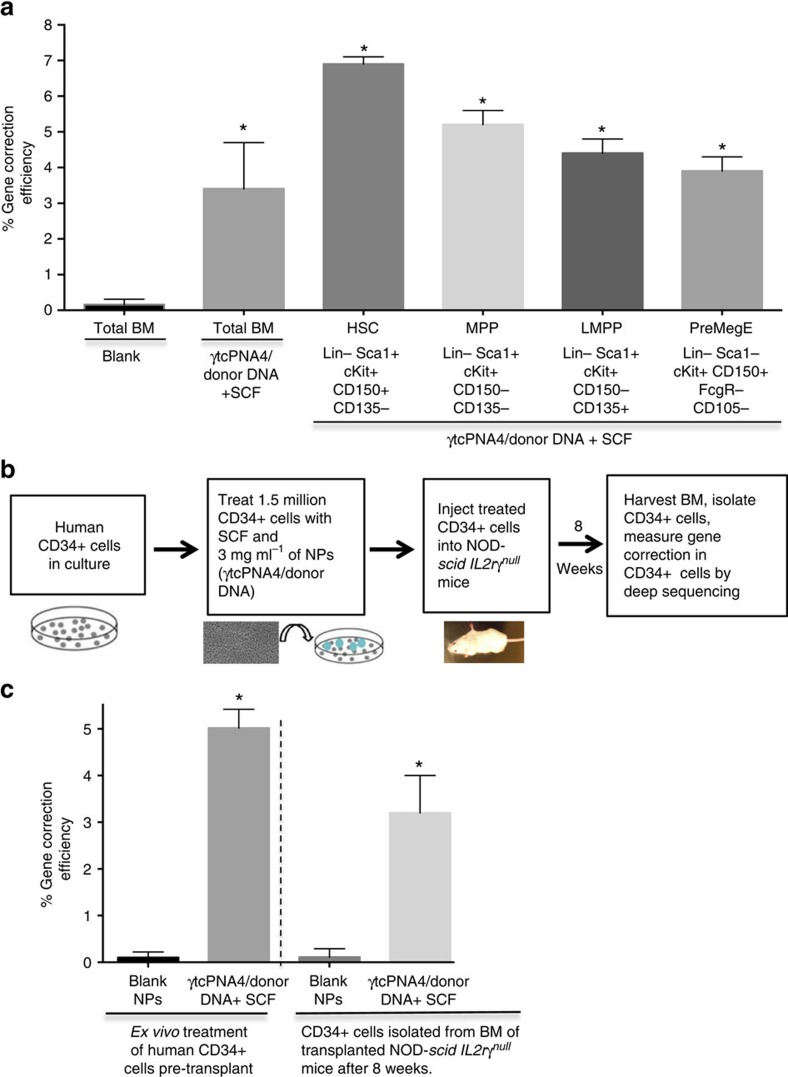
Gene editing in mouse BM stem cell populations *in vivo* and human CD34+ cells *ex vivo.* (**a**) Thalassemic mice were treated with either blank NPs or NPs containing γtcPNA4/donor DNA plus SCF (four doses at 2-day intervals as in Fig. 4). Deep sequencing analysis was performed to measure gene editing in the β-globin gene in either total BM (total BM) cells or in BM stem/progenitor cell sub-populations selected based on the indicated markers. BM cells were harvested either on day 36 post-treatment (total BM samples) or on day 65 post-treatment (sorted cell sub-populations). Data represent the combined analysis of BM from *n*=3 mice in each group. Data are mean±s.e.m., *n*=3; statistical analysis by Student's *t*-test, *P<0.05. (**b**) Schematic showing experimental design in which human CD34+ cells were treated *ex vivo* with either blank NPs or with γtcPNA4/donor DNA NPs plus SCF. Treated cells were either harvested 2 days later for deep sequencing analysis of gene editing in the β-globin gene or were transplanted into NOD-*scid IL2rγ*^*null*^mice. Eight weeks after transplantation, BM cells were harvested from the mice and human CD34+ cells were isolated, followed by deep sequencing of the the β-globin gene alleles. (**c**) Deep sequencing results to quantify β-globin gene editing in either pre-transplanted human CD34+ cells or in human CD34+ cells that were harvested from NOD-*scid IL2rγ*^*null*^ mice 8 weeks after transplant, as described in **b**. Data are mean±s.e.m., *n*=3 statistical analysis by Student's *t*-test, *P<0.05.

Deep sequencing analyses were performed on total BM cells of mice on day 36 post-treatment. Correction of the targeted mutation was seen at a frequency of almost 4% in the γtcPNA4/donor DNA treated group (Fig. 5a; combined analysis of 3 mice), whereas no correction was seen in the mice treated with blank NPs (Fig. 5a). Deep sequencing was also used to assess off-target effects in the BM cells at seven sites with partial homology to the binding site of γtcPNA4 in the β-globin gene. We found extremely low frequencies of off-target effects in the γtcPNA4/donor DNA-treated thalassemic mice (Table 2). The overall off-target modification frequency was 0.0032%, 1,218-fold lower than the frequency of β-globin gene editing.

In addition, we sorted the cells from the BM of the SCF and γtcPNA4/donor DNA NP treated mice (the BM was collected 65 days after the last treatment) for markers consistent with several stem/progenitor cell populations^23^, and we again performed deep sequencing of the β-globin gene. This revealed evidence for gene editing at a frequency of 6.9% in Lin-Sca1+ cKit+ CD150+ CD135-cells (Fig. 5a and Supplementary Fig. 20), a population that is highly enriched for long-term HSCs. We also observed gene editing in multiple other progenitor populations (Fig. 5a).

We also noted that the β-thalassemic animals had elevated levels of CD117+ cells in their BM compared with the phenotypically normal β-globin/GFP transgenic mice (Supplementary Fig. 21), indicative of the stress erythropoeisis in these animals. We also found that the proportion of CD117+ cells was further increased by SCF treatment. These elevated levels of CD117+ cells, and the stress erythropoeisis that underlies them, could explain, in part, the increased susceptibility to gene editing in the thalassemic mice.

### Gene editing by γtcPNAs in CD34+ human HSCs

We next tested the gene editing potential of γtcPNA4/donor DNA NPs in human CD34+ cells. Because we did not have cells from a thalassemic patient, we obtained human CD34+ cells from healthy donors from a cell bank. Consequently, we used a modified donor DNA designed to introduce a mutation at position IVS2-654 rather than correct it. One day after the CD34+ cells were thawed into stem cell medium, they were treated either with blank NPs or with NPs containing γtcPNA4/donor DNA plus SCF. Two days later, we performed deep sequencing, revealing β-globin gene editing at position IVS2-654 at a frequency of 5.0% (Fig. 5b,c and Table 3). Six off-target sites with partial homology to the binding site of γtcPNA4 in the β-globin gene were also analysed, and extremely low off-target frequencies were found in two of the sites sites (0.000017 and 0.000055%), with four sites showing no detectable sequence changes out of millions of reads (Table 3). Combined, we saw off-target mutations at an overall frequency of only 0.000012%, more than 400,000-fold lower than the 5% frequency of editing in the targeted β-globin gene. In addition, cytokine array analysis of supernatant taken from the treated CD34+ cells showed a minimal cytokine response (Supplementary Fig. 22).

We also performed a transplantation experiment in which human CD34+ cells that were treated *ex vivo* with γtcPNA4/donor DNA NPs and SCF were then transplanted into NOD-*scid IL2rγ*^*null*^mice. Eight weeks later, human CD34+ cells were isolated from the BM of the mice and deep sequencing was performed to measure the presence of gene editing in the β-globin gene (Fig. 5b). We found that 3.4% of β-globin gene alleles showed the introduced mutation at position IVS2-654 (Fig. 5c).

## Discussion

In the work reported here, we developed a gene editing strategy to correct a thalassaemia-associated mutation in the human β-globin gene and tested it in an established rodent model of human β-thalassaemia. Our results demonstrate that chemically modified, triplex-forming γPNAs and donor DNAs delivered intravenously via polymer NPs, and given in combination with SCF treatment, can mediate gene editing *in vivo* at a level sufficient to ameliorate the disease phenotype in the mice. We observed sustained reversal of the anaemia for up to 140 days post treatment, with normalization of serum haemoglobin concentrations and suppression of the reticulocytosis. We also saw a morphologic improvement in RBC cytology, along with reduced extramedullary haematopoiesis and reduction in splenomegaly. These findings suggest that our approach has the potential to yield a significant clinical response that could relieve the morbidity and mortality associated with β-thalassaemia.

There are two key advances for gene editing in this work. One is the incorporation of next generation PNA chemistry by substitution at the gamma position to enhance the binding properties of the PNAs. The second is the finding that the SCF/c-Kit pathway promotes increased gene editing by triplex-forming PNAs and donor DNAs. Upon *ex vivo* treatment of BM cells with γPNAs, the gene editing frequency in c-Kit+ cells was as high as 8%, 3–4-fold higher than with unmodified PNAs. The combination of SCF treatment with the γPNAs yielded even higher frequencies in the c-Kit+ cells, with just over 15% in a single treatment. *In vivo*, treatment of transgenic mice carrying a β-globin/GFP reporter transgene by i.p. injection of SCF followed by intravenous administration of NPs containing γPNAs and donor DNAs yielded gene editing in CD117+ cells at frequencies up to 1% in a single treatment. In the thalassemic mouse model, simple intravenous injection of γPNA/donor DNA NPs, plus SCF given i.p., resulted in a gene editing frequency of almost 4% in total BM cells (and 6.9% in Lin-Sca1+ cKit+ CD150+ CD135- cells, representing a sub-population of putative HSCs) and produced sustained amelioration of the disease phenotype, achieved in a minimally invasive manner without the need for stem cell harvest or transplantation.

Similarly, we achieved a frequency of 5% gene editing at the endogenous β-globin gene in human CD34+ haematopoietic progenitor cells treated once with SCF and γPNA/donor DNA NPs *ex vivo*, and we showed that these cells can engaft into NOD-*scid IL2rγ*^*null*^ mice with persistent evidence of gene editing, supporting the eventual translatability of our approach to individuals with thalassaemia.

Importantly, in a series of assays for haematopoietic colony formation, for induction of inflammatory cytokines, for generation of strand breaks and for off-target mutagenesis, there was essentially no measurable cellular toxicity and very low off-target effects from the γPNA-containing NPs in either the mouse or human cells. In the human CD34+ cells, the frequency of off-target mutations at the 6 most closely homologous sites was 0.000012%, a value more than 400,000-fold lower than the gene editing frequency of 5%, providing a possible safety advantage relative to other gene editing approaches^6^.

Two recent publications used either TALENs or CRISPR/Cas9 for gene editing in cell culture of human iPS cells carrying the same IVS2-654 thalassaemia-associated β-globin mutation site as studied here^24^,^25^. These publications achieved higher editing frequencies of 33% by TALENS and 12.3% by CRISPR/Cas9 in one report^24^ and 16.7% by CRISPR/Cas9 in the other^25^, compared with our editing frequency in human CD34+ cells of 5% (albeit a different cell type). With respect to off-target effects, these publications used a PCR-based T7E1 assay, and so quantitative comparisons with our deep sequencing results are not possible. Also, these publications did not test their approaches *in vivo*, and so we cannot make comparisons to our *in vivo* results in the thalassemic mice.

CD117 is the product of the c-Kit gene and is a receptor tyrosine kinase that mediates downstream signalling. Our results suggest that activation of this pathway promotes gene editing. Mechanistically, we observed elevated DNA repair gene expression in CD117 cells, including factors that may play a role in triplex-induced gene editing^4^,^10^,^26^,^27^. Importantly, we show that CD117+ cells show functional increases in DNA repair and that treatment with SCF produces further increases.

The 4% frequency of gene editing in total BM cells (and 6.9% in Lin-Sca1+ cKit+ CD150+ CD135- putative HSCs) achieved in the thalassemic mice was sufficient to achieve a clear improvement in phenotype. That gene correction at these frequencies could confer a phenotypic impact is consistent with transplantation studies in thalassemic mice and in patients in which mixed chimerism at one ratio of wild-type donor to thalassemic recipient cells in the marrow has produced much higher proportions of donor RBCs in the periphery^28^,^29^. This effect has been attributed to increased survival of genetically corrected erythroblasts during erythropoiesis, decreased ineffective erythropoiesis and increased survival in the circulation of corrected erythrocytes relative to thalassemic RBCs^30^.

Overall, our results provide motivation for further development of NP-mediated delivery of γPNAs and donor DNAs as a possible therapeutic strategy to achieve *in vivo* gene editing for treatment of human genetic disorders. The work here demonstrates gene editing in BM, but other work from our group has shown that NP delivery of PNAs to lung airway epithelia is also possible to achieve correction of the CFTR gene mutation associated with cystic fibrosis^31^,^32^. In this regard, the effect of SCF on gene editing in BM raises the possibility that other cytokines or growth factors could similarly serve to boost gene editing potential not only in BM but also in other tissues. In addition, the ability of SCF (which is an established activator of HSCs and is in clinical use) to stimulate gene editing by triplex-forming PNAs may also be applicable to other editing methods, such as CRISPR/Cas9.

Further improvements in PNA-mediated gene editing are still possible via additional PNA chemical modifications. Most encouraging at this point, however, is that even though γPNAs show improved gene editing potency, the off-target effects in the genome remain extremely low. This is in accordance with the lack of intrinsic nuclease activity in PNAs, and reflects the mechanism of triplex-induced gene editing, which creates an altered helix that engages endogenous DNA repair pathways.

## Methods

### Oligonucleotides

All ^MP^γPNA monomers were prepared from Boc-(2-(2-methoxyethoxy)ethyl)-L-serine as a starting material by a series of multistep synthetic procedures including reduction, mitsunobu reaction, nucleobase (A,C,G and T) conjugation and then ester cleavage. At each step the respective product was purified by column chromatography^20^. PNA oligomers were synthesized on solid support using Boc chemistry^16^. The oligomers were synthesized on MBHA (4-methylbenzhydrylamine) resin according to standard procedures of Boc chemistry. Kaiser test was performed at each step to measure complete coupling and double coupling was performed if it was required. The oligomers were cleaved from the resin using an m-cresol/thioanisole/TFMSA/TFA (1:1:2:6) cocktail, and the resulting mixtures were precipitated with ethyl ether, purified by reversed phase-high-performance liquid chromatography (acetonitrile:water) and characterized with a matrix-assisted laser desorption/ionization time-of-flight mass spectrometer.

The sequences of PNAs used in this study are given in Fig. 1. The single-stranded donor DNA oligomer was prepared by standard DNA synthesis except for the inclusion of three phosphorothiate internucleoside linkages at each end to protect from nuclease degradation. The sequence of the donor DNA matches positions 624–684 in β-globin intron 2 and is as follows, with the correcting IVS2-654 nucleotide underlined: 5′AAAGAATAACAGTGATAATTTCTGGGTTAAGGCAATAGCAATATCTCTGC ATATAAATAT3′.

For gene editing experiments in the human CD34+ studies, the donor DNA was designed to introduce rather than correct the IVS2-654 mutation, and so the sequence of the donor was:

5′-AAAGAATAACAGTGATAATTTC TGGGCGTTCTCAATAGCAATATCTCTGCATATAAATAT-3′. Note that besides the IVS2-654 mutation, this donor was designed to introduce 5 other point mutations adjacent to the IVS2-654 bp (underlined) to further facilitate detection by deep sequencing.

### PLGA nanoparticle synthesis and characterization

PLGA nanoparticles containing PNAs and DNAs were formulated using a double-emulsion solvent evaporation method and characterized PLGA nanoparticles encapsulating PNA/donor DNA were formulated using a double-emulsion solvent evaporation technique. PNAs and donor DNAs were dissolved in 60.8 μl DNAse-free water. All nanoparticle batches had 2 nmole mg^−1^ of PNA or γPNA and 1nmole mg^−1^ of donor DNA. The encapsulant was added dropwise to a polymer solution containing 80 mg 50:50 ester-terminated PLGA dissolved in dichloromethane (800 μl), then ultrasonicated (3 × 10 s) to formulate the first emulsion. To form the second emulsion, the first emulsion was added slowly dropwise to 1.6 ml of 5% aqueous polyvinyl alcohol and then ultrasonicated (3 × 10 s). This mixture was finally poured into 20 ml of 0.3% aqueous polyvinyl alcohol and stirred for 3 h at room temperature. Nanoparticles were then thoroughly washed with 20 ml water (3 × ) and further collected each time by centrifugation (12,000 r.p.m. for 10 min at 4 °C). Nanoparticles were resuspended in water, frozen at −80 °C, and then lyophilized. Nanoparticles were stored at −20 °C after lyophilisation^21^. Nucleic acid release was analysed by incubating nanoparticles (4–6 mg) in phosphate-buffered saline (PBS; 600 μl) in a 37 °C shaker, spinning down and removing supernatant. Further absorbance of the supernatant was measured at 260 nm at the indicated time points. At 24 and 48 h nanoparticles, the residual nucleic acid in the nanoparticle pellet was extracted and total nucleic acid content was calculated as a sum of absorbance obtained from the pellet as well as supernatant. Absorbances at 260 nm were measured with a Nanodrop 2,000 (ref. 21).

### *Ex vivo* experiments with BM cells from β-globin/GFP mice

BM cells were harvested by flushing femurs and tibias of β-globin/GFP transgenic mice with Roswell Park Memorial Institute (RPMI)/10% foetal bovine serum (FBS) media. Nanoparticles (2 mg ml^−1^) were used to treat ∼300,000–500,000 cells for 48 h in RPMI/10% FBS media containing glutamine, in triplicate samples. After 48 h, cells were fixed with 4% paraformaldehyde, and analysed by flow cytometry. Cells treated with blank nanoparticles were included as a control.

For experiments with CD117+, cells were isolated by magnetic separation and grown in cells, Iscove's Modified Dulbecco's Media media containing insulin (10 ng ml^−1^), FCS (10%) and erythropoietin (1 U ml^−1^). Where indicated, 3 μg ml^−1^ of SCF (recombinant murine SCF, catalogue #250-03, PeproTech, Rocky Hill, NJ;) was added before nanoparticle treatment. NPs ( 2 mg ml^−1^) were used to treat 50,000–100,000 CD117+ cells in triplicate for 48 h in the above media, followed by flow cytometry analyses as above. Inhibitors were used at concentrations of 200 nM (dasatinib), 1.0 μM (MEK162) and 3.0 μM (BKM120). Dasatanib was obtained from Cayman Chemical (Ann Arbor, MI; item #11498) and dissolved according to manfacturer's protocol. MEK162 and BKM120 were obtained from Dr. Harriet Kluger, Yale University.

### Sorting and flow cytometry of cells from β-globin/GFP mice

BD Bioscience kit catalogue #558451 (BDImag Haematopoietic Progenitor Stem Cell Enrichment Set—DM) was used to isolate CD117 cells. Enrichment for CD117 was confirmed by flow cytometry. CD117+ enriched cells were labelled with CD117-APC (BD Pharmingen catalogue #558451) antibody used a dilution of 1:100. Cells were co-labelled with control IgG antibody (BD Pharmingen catalogue #555746 used a dilution of 1:100) for gating purposes. To quantify GFP expression, after CD117 co-labelling, flow cytometry was performed using FACScaliburS by resuspending cells in PBS/1%FBS where green fluorescent cells are measured in the Fl1 channel and APC stained cells are in the Fl4 channel. Antibodies for other markers were Ter119 (BD Pharmingen catalogue #561033) and CD45 APC (BD Pharmingen catalogue #561018), both used a dilution of 1:100. See below for isolation of progenitor cell populations from thalassemic mice.

### DNA binding gel shift assays

For gel electrophoresis, synthetic 120 bp dsDNA targets were incubated with indicated oligomers at 37 °C in low ionic strength buffer (10 mM NaPi, pH 7.4). The samples were separated on 10% non-denaturing polyacrylamide gels in 1 × TBE buffer. The gels were run at 100 V cm^−1^ for 1.5 h. After electrophoresis, the gels were stained with 1 × SYBR-Gold (catalogue #S11494, Invitrogen) for 10 min, washed 2 × with 1 × TBE buffer, and then imaged using a gel documentation system (BioDoc-It System). The images were then inverted using Adobe Photoshop 6.0.

### Comet assay

400,000 BM cells per well were plated on 6-well plates in 1 ml media, then treated with 2 mg ml^−1^ of either blank NPs or PLGA NPs containing either tcPNA1/donor DNA, γtcPNA4/donor DNA or bleomycin/donor DNA, as indicated in Supplementary Fig. 4. After 48 h, cells were harvested, and prepared using the Trevigen Comet Assay kit per manufacturer's protocol (Trevigen, Gaithersburg, MD). Briefly, cells were suspended in agarose, added to comet slides, allowed to set, incubated 1 h in lysis solution, placed in electrophoresis solution for 30 min, then run at 21 V for 45 min, placed in acetate solution for 30 min, transferred to 70% ethanol solution for 30 min, dried, stained with Sybr Green for 30 min and then visualized using an EVOS microscope. TriTek Comet Score freeware was used to analyse images.

### Reporter gene assay for homology-dependent repair

An inactivating I-Sce1 site was cloned 56 amino acids into the firefly luciferase open reading frame under the control of a cytomegalovirus (CMV) promoter. The reporter construct also contains a promoterless luciferase gene used as a template for homologous recombination. A DSB in the luciferase reporter is created by *in vitro* digestion with the I-Sce I restriction enzyme (NEB # R0694L). Plasmid DNA was digested with I-Sce 1 for 1 h at 37 °C at a ratio of 10 units enzyme to 1 μg DNA and then the enzyme was inactivated at 65 °C for 20 min. The linearization of the plasmid was confirmed for each digestion via gel electrophoresis and the linear plasmid was purified using the Qiagen Qiaquick spin columns. After separation, CD117+ and CD117-cells from BM of β-globin/GFP transgenic mice, cells were transfected using the Lonza 2b Nucleofector Device. 5 × 10^5^ cells were transfected with 1 μg of either the luciferase reporter vector or a positive control firefly luciferase expression vector, along with 50 ng of a renilla luciferase expression plasmid as a transfection efficiency control. All transfections were performed in triplicate. After transfection the cells were plated at a density of 5 × 10^5^ cells per ml in 12-well plates. After 24 h incubation post transfection, luciferase activity was measured using the Promega Dual Luciferase Assay Kit. In each sample firefly luciferase activity was normalized to the renilla luciferase transfection control. Reporter reactivation was calculated as a ratio of normalized firefly luciferase activity in the cells transfected with the reporter plasmid to the positive control.

### Mouse models and *in vivo* treatments

All animal use was in accordance with the guidelines of the Animal Care and Use Committee of Yale University and conformed to the recommendations in the Guide for the Care and Use of Laboratory Animals (Institute of Laboratory Animal Resources, National Research Council, National Academy of Sciences, 1996).

The β-globin/GFP transgenic mice were obtained from Ryszard Kole, University of North Carolina^19^. For treatment of the mice, where indicated SCF (220 μg kg^−1^ per mouse, Recombinant Mouse SCF, carrier-free, R&D catalogue #455-mc-050/CF) was injected intraperitoneally 3 h before treatment with 4 mg of NPs in 150 μl PBS delivered via retro-orbital intravenous injection. In some cases, mice were killed 48 h after the NP injections, and BM and spleen cells were harvested for further analysis. The BM and spleen cells (500,000 each) were co-labelled with APC conjugated antibodies as described above and flow cytometry was performed as above. For deep sequencing analyses, CD117+ cells were isolated by magnetic separation using a BD Bioscience protocol (BDImag Hematopoietic Progenitor Stem Cell Enrichment Set—DM). Genomic DNA from three mice was pooled followed by sequence analysis as previously described^12^.

The IVS2-654 β-thalassemic mice were obtained from Ryszard Kole, University of North Carolina^18^. For *in vivo* experiments, where indicated, SCF (220 μg kg^−1^ per mouse, Recombinant Mouse SCF, carrier-free, R&D catalogue #455-mc-050/CF) was injected intraperitoneally 3 h prior to treatment with 4 mg of NPs in 150 μl of PBS delivered via retro-orbital intravenous injection. Each mouse received four treatments given at 48 h intervals. Mice were anesthetized with isoflurane followed by retro-orbital bleeding (∼100 μl) using ethylenediaminetetraacetic acid–treated glass capillary tubes. The blood was evacuated into tubes with 5 μl of 0.5 M EDTA acid in heparinized coated tubes. Complete blood counts were performed using a Hemavet 950FS (Drew Scientific, Oxford, CT) according to the manufacturer's protocol. Slides containing blood smears were stained with Wright and Giemsa stain for microscopy. Methylene blue staining was used for reticulocyte counts. Spleen images and weights were taken after selected mice were killed on day 36 after the last treatment. Harvested spleens were fixed in 10% neutral buffered formalin and processed by Yale Pathology Tissue Services for H&E, CD61 and E cadherin staining.

For assigning animals into treatment groups as listed above, littermate animals were genotyped, and then the pups carrying the required genotypes (either β-globin/GFP transgenic mice or IVS2-654 β-thalassemic mice) were randomized into the several treatment groups in cohorts of 3–6, as indicated, at 8 weeks of age. Both genders were included. The investigators were not blinded as to treatment groups.

### Deep sequencing analyses

Genomic DNA from mouse cells treated *ex vivo* or from cell from mice treated *in vivo,* as indicated, was harvested using the Wizard Genomic Purification Kit (Promega), and then electrophoresed in a 1% low melting point agarose gel in TAE, to separate genomic DNA from possible residual PNA and/or DNA oligonucleotide. The high-molecular weight species, representing genomic DNA, was cut from the agarose gel and extracted using the Wizard SV Gel and PCR Clean-Up System (Promega) according to manufacturer's instructions. Once genomic DNA was isolated from treated cells or mouse tissue, PCR reactions were performed with high fidelity TAQ polymerase. Each PCR tube consisted of 28.2 μl dH_2_O, 5 μl 10 × HiFi Buffer, 3 μl 50 mM MgCl_2_, 1 μl DNTP, 1 μl each of forward and reverse primer, 0.8 μl High Fidelity Platinum Taq Polymerase (Invitrogen, Carlsbad, CA) and 10 μl DNA template. PCR products were prepared by end-repair and adaptor ligation according to Illumina protocols (San Diego, CA), and samples sequenced by the Illumina HiSeq with 75 paired-end reads at the Yale Center for Genome Analysis. Samples were analysed as previously described^12^. Primers for deep sequencing were designed using Primer3 database. The primers used for β−globin intron 2 were as follows: forward primer: 5′-TATCATGCCTCTTTGCACCA-3′; reverse primer: 5′-AGCAATATGAAACCTCTTACATCA-3′. Primers for off-target sites of partial homology were as follows; forward primer is listed first: vascular cell adhesion protein precursor 1 (5′-AGATAATTATTGCCTCCCACTGC-3′ and 5′-AATGGAAGGGCATGCAGTCA-3′); polypyrimidine tract binding protein (5′-CCCAATCCTGAATCCTGGCT-3′ and 5′-CATACTGATGTCTGTGGCTTGA-3′); protocadherin fat 4 precursor (5′-AAGCTCAAACCTACCAGACCA-3′ and 5′-AGCTGGAAGCTTCTTCAGTCA-3′); olfactory receptor 266 (5′-CCCTCTGTGGACTGAGGAAG-3′ and 5′-TGATGAGCTACGGGTATGTGA-3′); syntaxin binding protein (5′-CAAAAAGCCTTAAGCAAACACTC-3′ and 5′-TCTCTCCCTCAGCATCTATTCC-3′); muscleblind-like protein (5′-TGTGTTTGTTTATGGATACTTGAGC-3′ snd 5′-GCATGCACAATAAAGGCACT-3′); ceruloplasmin isoform (5′-CATGGGAAACAGTCAAAAGAAA-3′ and 5′-TGTAGGTTTCCCCACAGCTT-3′).

### Cytokine array analysis

NPs (4.0 mg) containing γtcPNA4/donor DNA were injected i.v. into β-globin/GFP transgenic mice. After, and after 48 h, plasma samples were collected. Cytokine array analyses were performed on 25.0 μl of plasma and analysed for cytokines using the luminex based cytokine detection and quantification technology at the CytoPlex Core Facility at Yale University (https://medicine.yale.edu/obgyn/drs/immunology/).

### Analysis of HSCs from BM of treated thalassemic mice

Thalassemic mice were treated as above with four treatments of NPs atat 2 day intervals of 4 mg of NPs in 150 μl PBS delivered via retro-orbital intravenous injection along with SCF given i.p. Sixty-five days later, mouse tibias and femurs were collected and crushed in mortar with 5 ml fluorescence-activated cell sorting (FACS) buffer (PBS+0.5% bovine serum albumin+2 mM EDTA). The BM was passed through a 100 μ cell strainer and then the cells were collected by centrifugation at 250*g* for 5 min at room temperature. The cell pellets were resuspended in 5 ml of RBC lysis buffer for 1 min. The suspensions were diluted with 1 ml FBS and 14 ml FACS buffer, cells were collected by centrifugation, and then resuspended in FACS buffer for antibody staining, followed by FACS. Antibodies used were as follows: from BD Biosciences (www.bdbiosciences.com): BD553086 (CD45R/B220), BD553309 (CD11b), BD553672 (Ter119), BD553060 (CD3e), BD553028 (CD8a), BD553649 (CD4); BD562729-Ly-6A/E (Sca1), BD565502-CD16/CD32 (FcgR); from BioLegend (http://www.biolegend.com): APC c-Kit (BioLegend 105812-CD117), CD105 (BioLegend 120406-CD105), PE-Cy5CD135 (BioLegend 135312-CD135), PE-CD150 (BioLegend 115904-CD150 SLAM), PE/Cy7 Streptavidin (BioLegend 405206-Biotin). Antibodies were used at 0.2–0.8 μg ml^−1^. From isolated cell populations, genomic DNA extraction was performed using the Wizard Genomic Purification Kit (Promega) and samples were submitted for deep sequencing as above.

### PCR with reverse transcription analysis

Cells were harvested, pelleted and frozen in RNA stabilization reagent (Qiagen), until ready for RNA extraction. RNA was extracted from the cell pellets using the RNAeasy Mini Plus kit from Qiagen, as per the manufacturer's protocol. The Invitrogen SuperScript III kit was used to generate cDNA from the RNA, as per the manufacturer's protocol, using 500 ng of RNA per reaction. PCR reactions contained cDNA, 20% Betaine, 0.2 mM dNTPS, Advantage 2 Polymerase Mix, 0.2 μM of each primer, 2% Platinum Taq, and Brilliant SYBR Green. Primers and ROX reference dye were obtained from Stratagene and analysis was conducted using a Mx3000p realtime cycler. Cycler conditions were 94 °C for 2 min, 40 cycles of 94 °C 30 s/50 °C 30 s/72 °C 1 min, then 95 °C for 1 min. Relative expression were calculated using the 2^ΔΔCt^ method (Ct<36) and then normalized. Mouse BRCA2 primers were designed using Primer3 database: BRCA2-3F: 5′-GTTCATAACCGTGGGGCTTA-3′ and BRCA2-3R: 5′-TTGGGAAATTTTTAAGGCGA-3′. For BRCA2 data analysis GAPDH were used as control using following primers: 5′-TGATGACATC AAGAAGGTGGTGAAG-3′ and 5′-TCCTTGGAGG CCATGTGGGCCAT-3′. For RAD51 analysis, Rad51 mRNA was quantified by using TaqMan Gene Expression Assay (Life technologies, Mm00487905_m1) kit and using gene 18S (Life technologies, Mm03928990_g1) as a control.

### Western blot analysis

CD117+ and CD117-cells were isolated from β-globin/GFP mice and protein was extracted with Radio-Immunoprecipitation Assay lysis buffer. Total protein of 50–100 μg was run on SDS/polyacrylamide gel electrophoresis gels and transferred to nitrocellulose membranes. Antibodies used were: Anti-BRCA2 (Ab-1) mouse mAb (EMD Millipore, OP95-100 ug) at 1:10,000 anti-RAD51-antibody (Santa Cruz biotechnology, SC 8349) at 1:10,000.

### Microarray analysis

Microarray analyses were performed on CD117+ and CD117-cells obtained from BM of three separate β-globin/GFP mice at Yale Center of genomic analysis at Yale west campus. Each replicate cell sample was obtained from a separate mouse. RNA was extracted from 2 × 10^6^ for each sample using the RNeasy Mini Plus kit from Qiagen, as per the manufacturer's protocol. Following DNase treatment, total RNA was sequenced and analysed at the Yale Center for Genome Analysis. Heat maps were generated using variance stabilizing transformations of the count data on the basis of a parametric fit to the overall mean dispersions.

### γH2AX foci experiments

Samples of 20,000 BM cells each from β-gobin/GFP mice were either untreated or were treated with 2 mg ml^−1^ of NPs containing γtcPNA4 and donor DNA or with 5 Gy IR, as indicated in Supplementary Fig. 4F. After 48 h, cells were washed with PBS, fixed in 70% ice-cold ethanol, washed again with PBS and incubated for 15 min on ice in PBST buffer (PBS containing 1% bovine serum albumin and 0.2% Triton X-100). Cells were collected by centrifugation and incubated with an anti-phospho-H2AX antibody (Cell Signaling, #9718) diluted at a ratio of 1:200 in PBST buffer overnight at 4 °C. Cells were washed and incubated with an anti-rabbit antibody conjugated with Alexa 488 (Cell Signaling, #4412) diluted at a ratio of 1:100 in PBST buffer, and incubated for 1 h in the dark at room temperature. Cells were washed and resuspended in PBS for flow cytometry analysis.

For the primary fibroblasts from the β-globin/GFP mice, 50 × 10^3^ cells were pretreated with the indicated reagents in Fig. 1h,i for 48 h in chamber well slides (Millipore Millicell EZ Slides). Selected samples were irradiated with 5 Gy IR as a positive control. Cas9 and guide RNA (designed to bind to the same site in β-globin gene as γtcPNA4) expression plasmids were obtained from GeneCopoeia (www.genecopoeia.com). Cas9 and guide RNA expression plasmids were transfected using lipofectamine (Thermo Fisher Scientific; www.thermofisher.com), per manufacturer's instructions, either as separate expression vector plasmids (Cas9+gRNA) or contained on the same plasmid (Cas9 and gRNA). Cas9 and gRNA of 1.3 μg were added into each well for 48 h, as indicated. Cells were fixed with 1% paraformaldehyde/2% sucrose for 15 min at room temperature, followed by 100% methanol for 30 min at −20 °C and 50% methanol/50% acetone for 20 min at −20 °C. Slides were then incubated in permeabilization/blocking solution (10% BGS, 0.5% Triton X-100 in PBS) at room temperature for 1 h. Primary antibody (phospho-specific H2A.X (Cell Signaling #9718S)) was diluted 1:500 in permeabilization/blocking solution and used to stain cells at 4 °C overnight. The secondary antibody used was Alexa Fluor 488-conjugated goat anti-rabbit immunoglobulin G (IgG; Life Technologies). Cells were costained with DAPI to visualize the nuclei.

### Cell cycle analysis

Pre-sorted CD117 cells were treated with SCF for 48 h and then fixed in 70% ethanol, treated with 100 mg ml^−1^ RNase, and stained with propidium iodide. Cells were analysed by flow cytometry using 10,000 cells per condition.

### Treatment and analysis of human CD34+ cells

Human CD34+ cells were obtained from the Yale Center of Excellence in Molecular Hematology (Yale University, New Haven, CT) from granulocyte colony-stimulating factor-mobilized peripheral blood of normal healthy donors. Cells were received frozen, thawed, pooled and maintained in StemSpan serum-free expansion medium (SFEM) with StemSpan CC100 cytokine mixture (Stemcell Technologies, Vancouver, British Columbia, Canada). Cell counts were performed with a Nexcelcom Cellometer Auto T4 (Bioscience, Lawrence, MA). CD34 cells (6 × 10^5^) were treated with SCF (1.0 μg) and 2.0 mg of nanoparticles containing γtcPNA4/donor DNA in the SFEM with the CC100 cytokine mixture for 48 h. Cells were harvested, and genomic DNA extraction was performed using the Wizard Genomic Purification Kit (Promega). Samples were submitted for deep sequencing as above. For off-target analyses for the human DNA, the primers used were: serine threonine kinase (5′-TTTCTTGCCATGTTGGTGTG-3′ and 5′-CCTCCGGTCCTATTTGTTCA3′), anoctamin-3 (5′-TTGCATTTATTGGCAGCTTT3′□and 5′-TTCAGTGATTAAATTCTGTCACTCTG3′), 39s Ribosomal protein L17 (5′-TGCACTTATTAATCACCAACTCTG3′ and 5′-TGGCCCTTTGATATAGCTGTG3′), neuroblast differentiation associated protein (5′-AGGTGGGCAACATCAATTTC3′ and 5′-CAGGCCCAGCATCTTGTATT3′), transciption enhancer factor TEF1 (5′-TGCCAACACAGTGCTTTCTC3′ and 5′-GAGCTTTGTGAAGGCAGGAC3′), RhoGTPase activating protein (5′-AAGCTAAACGGTGTCTCTTTCTG3′ and 5′-GCATCATGGATCTGATTTGC3′).

For cytokine array analysis, after NP treatment of the CD34+ cells, as above, supernatant medium was collected and analysed by a luminex based assay as describe above.

### Transplantation of treated human CD34+ cells

Human CD34 cells (6 × 10^5^) were treated, as above, with SCF (1.0 μg) and 2.0 mg of nanoparticles containing γtcPNA4/donor DNA in the SFEM with the CC100 cytokine mixture for 48 h and then were used to transplant immunodeficient recipient NOD-*scid IL2rγ*^*null*^ mice. As above, all animal use was in accordance with the guidelines of the Institutional Animal Care and Use Committees (IACUC) of the University of Massachusetts Medical School, Yale University, and The Jackson Laboratory. NOD.Cg-*Prkdc*^*scid*^*IL2rγ*^*tm1Wjl*^ (abbreviated NOD-*scid IL2rγ*^*null*^) mice were obtained from the research colony maintained at The Jackson Laboratory. Transplant of treated CD34 cells into the NOD-*scid IL2rγ*^*null*^ mice was performed by intracardiac injection into newborn mice as previously described^12^. Engraftment was confirmed at 7 weeks. Eight weeks post transplant, human CD34+ cells were isolated from mouse BM using EasySep human CD34+ positive selection Kit (StemCell Technologies; Cat# 18056). Genomic DNA was extracted from CD34+ cells using the Wizard Genomic Purification Kit (Promega). Samples were submitted for deep sequencing as described above.

### Data availability

Microarray data have been deposited in Gene Expression Omnibus (GEO) database (http://www.ncbi.nlm.nih.gov/geo/) under accession code GSE86859.

## Additional information

**How to cite this article:** Bahal, R. *et al*. *In vivo* correction of anaemia in β-thalassemic mice by γPNA-mediated gene editing with nanoparticle delivery. *Nat. Commun.* **7,** 13304 doi: 10.1038/ncomms13304 (2016).

**Publisher's note:** Springer Nature remains neutral with regard to jurisdictional claims in published maps and institutional affiliations.

## Supplementary Material

Supplementary Information Supplementary Figures 1-24 and Supplementary Table 1.

## Figures and Tables

**Table 1:** Off-target effects in bone marrow cells following intravenous treatment of β-globin/GFP mice with γtcPNA4/donor DNA NPs.

**Gene locus**	**Sequences of partial homology (5′–3′)**	**Size of region sequenced**	**Alleles sequenced**	**Number modified**	**Frequency (%)**
β-globin	TGCCCTGAAAGAAAGAGA	128	1,399,786	78,833	0.56
Vascular cell adhesion protein precursor 1	AGCCCTGAAAGAAAGAGA	111	480,013	0	0
Polypyrimidine tract binding protein	GAACCTGAAAGAAAGAGA	101	349,723	26	0.0074
Protocadherin fat 4 precursor	CACCCTGAAAGAAAGAAG	115	73,245	0	0
Olfactory receptor 266	AAGCCTGAAAGAAAGATT	172	1,092,990	2	0.00018
Syntaxin binding protein	AGAAATGAAAGAAAGAGA	150	2,478,636	0	0
Muscleblind like protein	GGTGGTGAAAGAAAGAGA	165	2,331,971	0	0
Ceruloplasmin isoform	AGGACTGAAAGAAAGAGT	154	1,390,439	0	0
Total off-target			8,197,017	28	0.00034

The top seven gene loci in the mouse genome with partial homology to the 18 bp γtcPNA4 target site in β-globin intron 2 were identified, with the sequences as indicated. β-globin/GFP mice were treated with SCF followed by intravenous infusion with NPs containing γtcPNA4/donor DNA, and genomic DNA from c-Kit+ BM cells was subject to deep sequencing analysis at these loci. The size of the region sequenced around each site is listed, along with the number of alleles sequenced and the number of alleles with modified sequences.

**Table 2:** Off-target effects in bone marrow cells following intravenous treatment of β-thalassemic mice with SCF and γtcPNA4/donor DNA NPs.

**Gene locus**	**Sequences of partial homology (5′–3′)**	**Size of region sequenced**	**Alleles sequenced**	**Number modified**	**Frequency (%)**
β-globin	TGCCCTGAAAGAAAGAGA	128	8,615,313	337,192	3.9
Vascular cell adhesion protein precursor 1	AGCCCTGAAAGAAAGAGA	111	482,051	0	0
Polypyrimidine tract binding protein	GAACCTGAAAGAAAGAGA	101	355,567	2	0.00056
Protocadherin fat 4 precursor	CACCCTGAAAGAAAGAAG	115	123,158	0	0
Olfactory receptor 266	AAGCCTGAAAGAAAGATT	172	1,099,880	262	0.0231
Syntaxin binding protein	AGAAATGAAAGAAAGAGA	150	2,493,024	0	0
Muscleblind like protein	GGTGGTGAAAGAAAGAGA	165	2,336,715	0	0
Ceruloplasmin isoform	AGGACTGAAAGAAAGAGT	154	1,397,271	0	0
Total off-target			8,287,666	268	0.0032

The top seven gene loci in the mouse genome with partial homology to the 18 bp γtcPNA4 target site in β-globin intron 2 were identified, with the sequences as indicated. Thalassemic mice were treated with SCF followed by intravenous infusion with NPs containing γtcPNA4/donor DNA, and genomic DNA from c-Kit+ BM cells was subject to deep sequencing analysis at these loci. The size of the region sequenced around each site is listed, along with the number of alleles sequenced and the number of alleles with modified sequences.

**Table 3:** Deep sequencing analysis of targeted gene editing versus off-target effects in human CD34+ haematopoietic cells following *ex vivo* treatment with SCF and γtcPNA4/donor DNA NPs.

**Gene locus**	**Sequences of partial homology (5′–3′)**	**Size of region sequenced**	**Alleles sequenced**	**Number modified**	**Frequency (%)**
β-globin	TGCCCTGAAAGAAAGAGA	128	12,489,910	606,230	5.02
Serine Theronine Kinase	ATTCCTGAAAGAAAGCAC	189	3,330,879	0	0
Anoctamin-3	AATTCTGAAAGAAAGACC	150	5,805,518	1	0.000017
39s Ribosomal protein L17	AGCCCTGAAAGAATACCA	169	4,211,251	0	0
Neuroblast differentiation associated	TCCCTGAAAGAAAAAAGA	198	3,579,389	2	0.000055
Transcription enhance factor TEF1	TCTCCCTGAAAGAAAAAA	244	80,548	0	0
Rho GTPase activating protein	CAACATGAAAGAAAGAGA	154	8,256,220	0	0
Total off-target			25,263,805	3	0.000012

The top six gene loci in the human genome with partial homology to the 18 bp γtcPNA4 target site in β-globin intron 2 were identified, with the sequences as indicated. Human CD34+ haematopoietic cells were treated *ex vivo* with SCF and with NPs containing γtcPNA4/donor DNA, and 2 days later genomic DNA from the cells was subject to deep sequencing analysis at these loci. The size of the region sequenced around each site is listed, along with the number of alleles sequenced and the number of alleles with modified sequences.
