# Supplementary material for: In vivo correction of anaemia in β-thalassemic mice by γPNA-mediated gene editing with nanoparticle delivery
Source: Nat Commun. 2016 Oct 26;7:13304. doi: 10.1038/ncomms13304 (PMC5095181; doi:10.1038/ncomms13304)
Supplement: Supplementary Information — Supplementary Figures 1-24 and Supplementary Table 1. [file ncomms13304-s1.pdf]

Supplementary Fig. 1

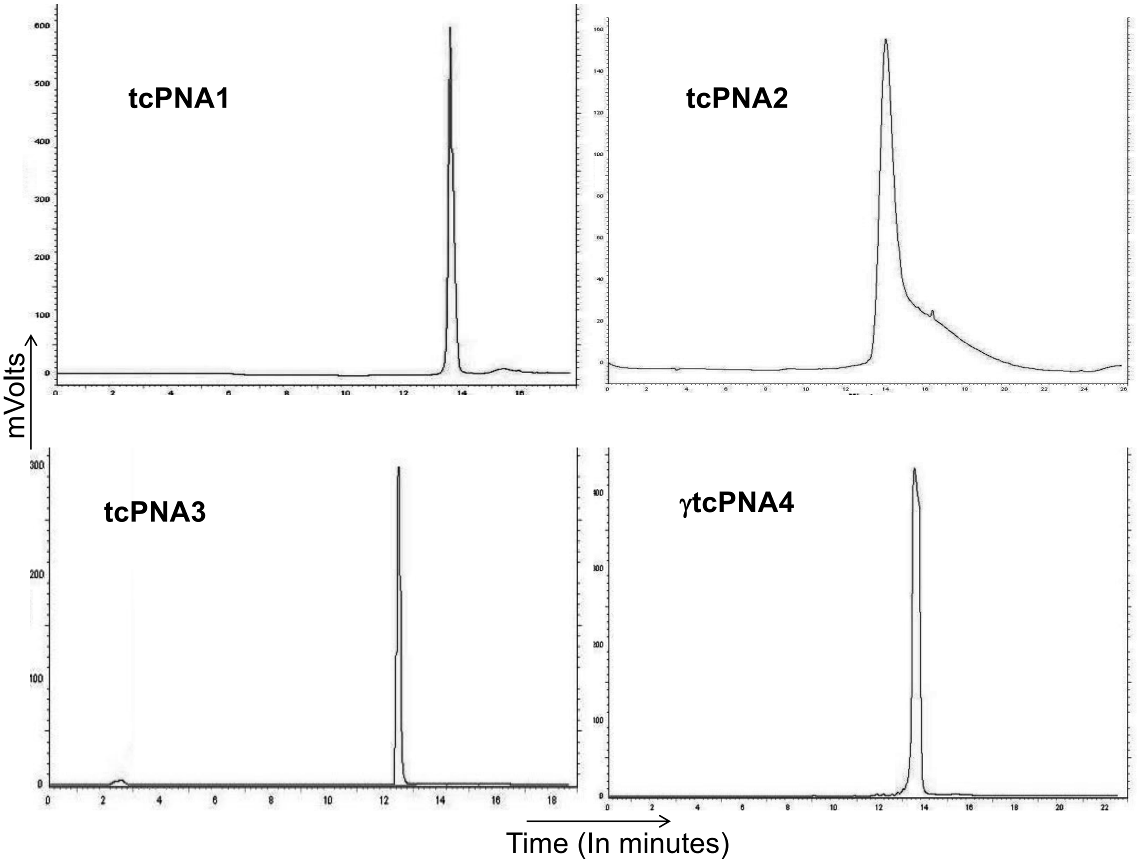

Supplementary Fig. 1. HPLC traces of purified PNAs using reverse phase HPLC. (Related to Fig. 1.) Water and acetonitrile containing 0.1% trifluoroacetic (TFA) acid were used as a solvent system. These tracings provide characterization of the purity of the synthesized PNAs and so were not subjected to statistical analysis.

Supplementary Fig. 2

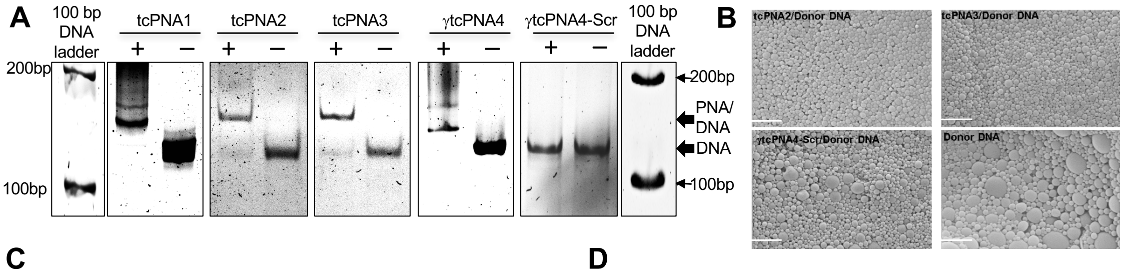

C

Sample	Diameter (nm)
tcPNA1/donor DNA	293.1 ± 6.1
tcPNA 2/donor DNA	610.6 ± 27.7
tcPNA 3/donor DNA	373.0 ± 4.3
γtcPNA 4/donor DNA	291.0 ± 4.7
γtcPNA 4-Scr/donor DNA	458.6 ± 8.2
Donor DNA	907.3 ± 200

D

Sample	Zeta Potential (mV)
tcPNA1/donor DNA	-24.6 ± 0.4
tcPNA2/donor DNA	-16.5 ± 0.5
tcPNA3/donor DNA	-23.6 ± 0.5
γtcPNA4/donor DNA	-23.4 ± 0.5
γtcPNA 4-Scr/donor DNA	-19.5 ± 1.3
Donor DNA	-29.1 ± 0.4

E

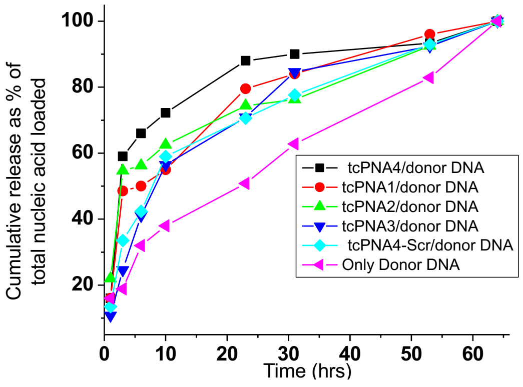

Supplementary Fig. 2. Evaluation of PNA and nanoparticle reagents. (Related to Fig. 1.) (A) Gel mobility shift assays demonstrating binding of tcPNA1, tcPNA2, tcPNA3, γtcPNA4 but not γtcPNA4-Scr to 120 bp double-stranded DNA fragments containing β-globin intron 2 sequences. Each 120 bp dsDNA contains the binding site for the respective tcPNA. Note: Double-stranded DNA substrates used for γtcPNA4 and γtcPNA4-Scr are the same. (B) Scanning electron microscope (SEM) images of PLGA nanoparticles containing tcPNA/donor DNA. Scale bar, 2.0 μm. (C) Hydrodynamic diameter of formulated PLGA nanoparticles measured using dynamic light scattering in PBS buffer. Data are presented as mean ± s.e.m., n = 3. (D) Zeta potential of formulated PLGA nanoparticles. Data are presented as mean ± s.e.m., n = 3. (E) Release of total nucleic acids from PLGA nanoparticles during incubation at 37°C in PBS. At 64 hrs, the residual nucleic acid in the NP pellet was extracted and the total nucleic acid load was calculated as a sum of absorbance obtained from the pellet and supernatant.

Supplementary Fig. 3

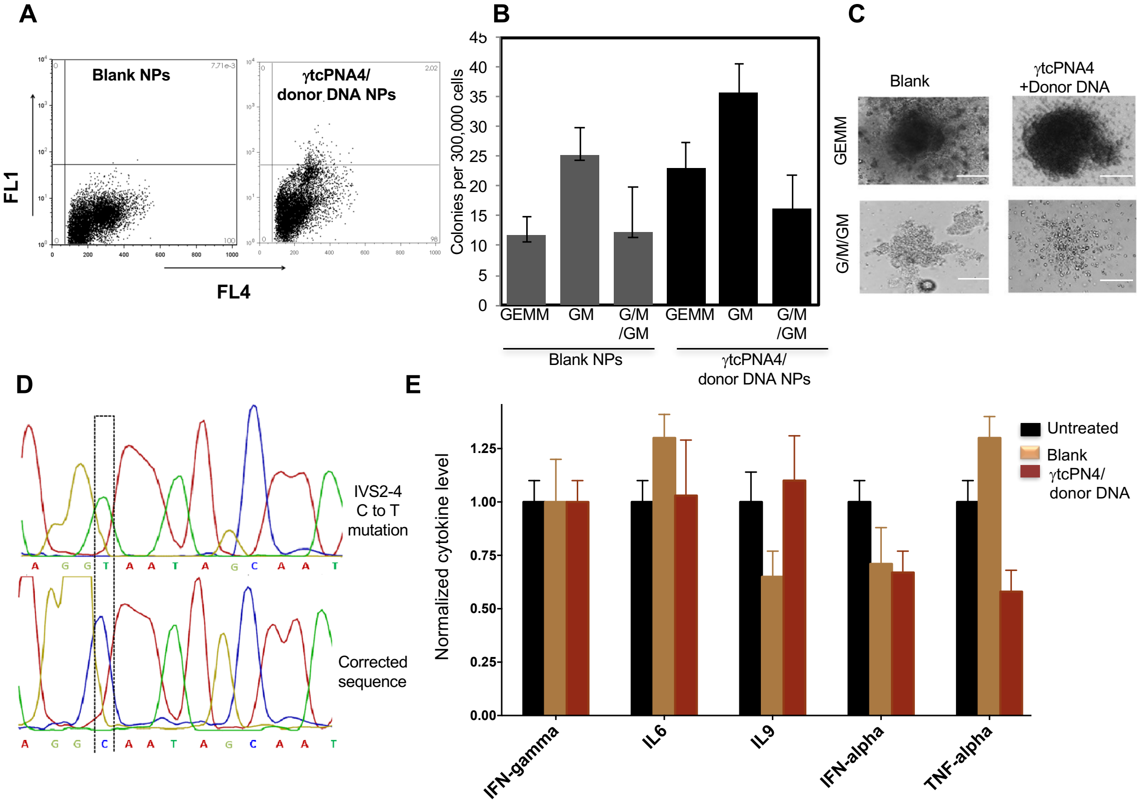

Supplementary Fig. 3. Gene editing of the IVS2-654 (C→ T) mutation in the human β-globin intron 2 sequences embedded within GFP in the β-globin/GFP fusion transgene: examples of primary data and lack of toxicity. (Related to Figs. 1 and 2). (A) Flow cytometry dot plots of total bone marrow cells analysed after treatment with γtcPNA4/donor DNA NPs as compared to blank NPs. GFP expression is scored in channel FL1. (B) Mouse total bone marrow cells were treated with either blank NPs or NPs containing γtcPNA4 and donor DNA and were plated for a colony-forming cell assay in methylcellulose medium with selected cytokines for growth of granulocyte/macrophage colonies (CFU-G, CFU-M and CFU-GM) or combined colonies (CFU-GEMM, granulocyte, erythroid, monocyte/macrophage, megakaryocyte. Numbers of each type of colony per 300,000 plated cells are shown. Data are shown as mean ± s.d., n = 3. (C) Representative images of hematopoietic colonies arising from treated bone marrow cells plated in methylcellulose media after treatment with γtcPNA4/donor DNA NPs. Scale bar, 20.0 µm. (D) Example of direct genomic sequencing analysis of colonies arising from bone marrow cells after treatment *ex vivo* with γtcPNA4/donor DNA containing PLGA nanoparticles. (E) Analysis of cytokine levels, as indicated, in bone marrow cells derived from GFP mice either untreated or treated with blank NP or γtcPNA4 and donor DNA NPs at 48 h post treatment. Data are presented as mean ± s.e.m., n = 3.

Supplementary Fig. 4

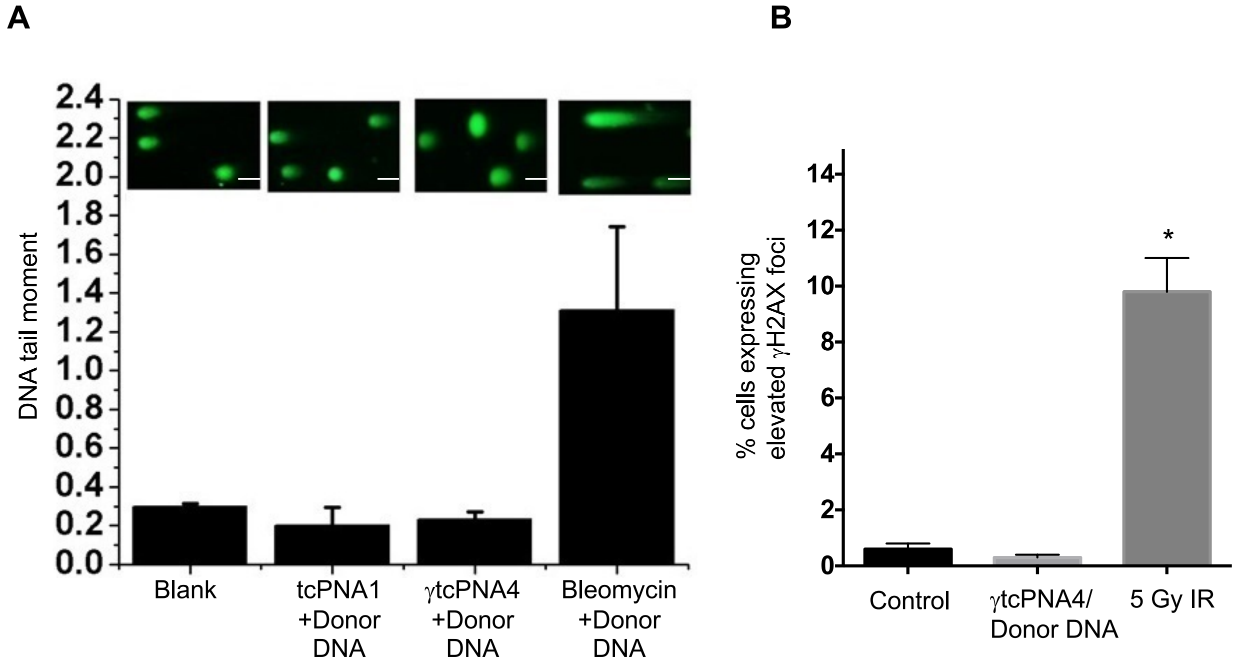

Supplementary Fig. 4. Comet and γH2X foci experiments to measure DNA DSBs. (A) Comet assay to measure DNA breaks in NP-treated bone marrow cells. Cells were treated with blank NPs or NPs containing either tcPNA1/donor DNA, γtcPNA4/donor DNA, or bleomycin/donor DNA, as indicated. DNA tail moment provides a measurement of the extent of breaks. Scale bar, 100.0 µm. (B) Quantification of immune fluorescent γH2AX foci by flow cytometry analysis in mouse bone marrow cells either untreated or treated with γtcPNA4/donor DNA NPs or with 5 Gy of ionizing radiation (IR), as indicated. Data are mean ± s.e.m., n = 3; analysis by student's t-test, $*p < 0.05$.

Supplementary Fig. 5

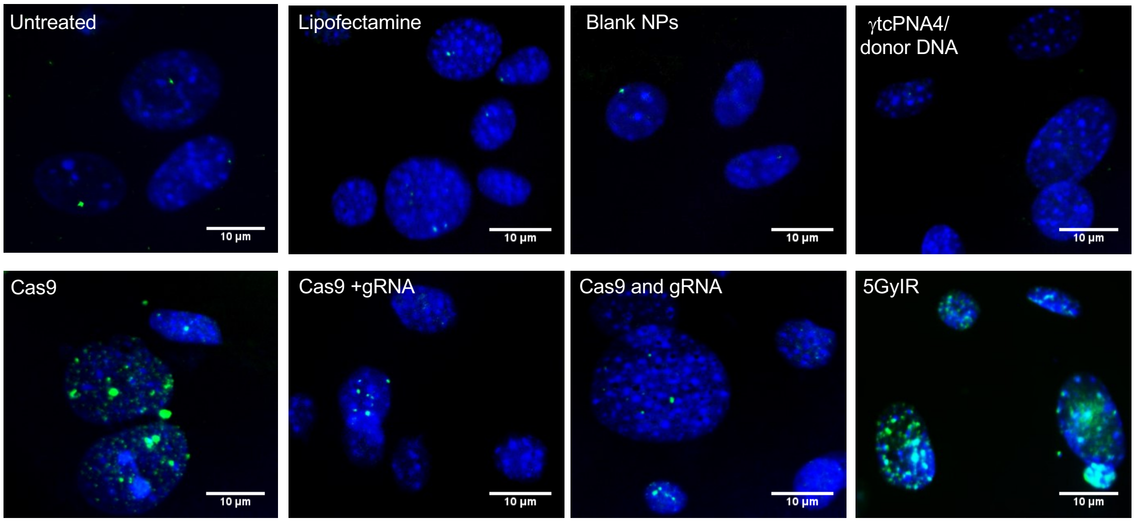

Supplementary Fig. 5. γH2AX foci. (Related to Fig. 1.) Representative images of γH2AX foci as a measure of DNA double-strand breaks in mouse cells after treatment with the indicated reagents, either untreated, exposure to lipofectamine transfection reagent alone, blank NPs, NPs containing γtcPNA4/donor DNA, a vector expressing the Cas9 nuclease and a separate vector expressing a guide RNA designed to bind to the same sequence in the β-globin gene as γtcPNA4 (Cas9 + gRNA), a single vector expressing both the Cas9 nuclease and the gRNA (Cas9 and gRNA), or 5 Gy or ionizing radiation (IR). Scale bars, 10.0 µm.

Supplementary Fig. 6

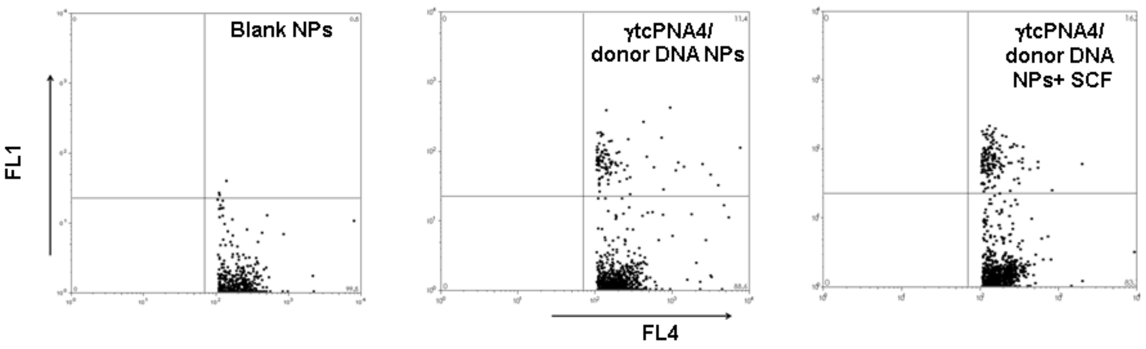

Supplementary Fig. 6. Representative flow cytometry dot plots. (Related to Fig. 2.) Flow cytometry dot plots of CD117+ cells after treatment with blank NPs, with NPs containing γtcPNA4/donor DNA, or with stem cell factor (SCF) followed by NPs containing γtcPNA4/donor DNA. GFP expression is scored in channel FL1.

Supplementary Fig. 7

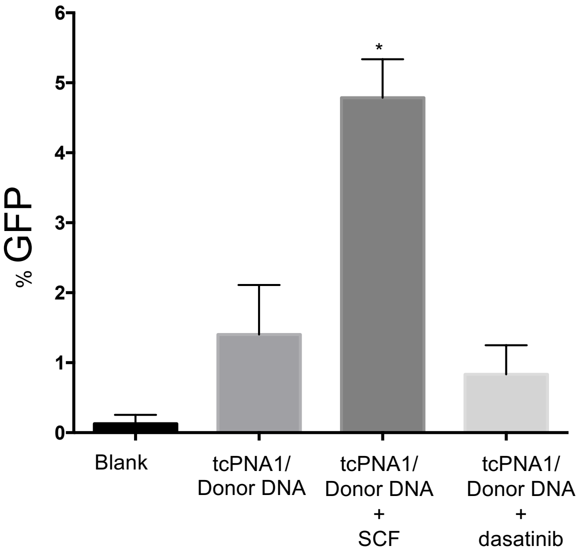

Supplementary Fig. 7. Gene editing by tcPNA1 in pre-sorted CD117 cells. (Related to Fig. 2.) %GFP expressing CD117+ cells from β-globin/GFP transgenic mice after *ex vivo* treatment with NPs containing tcPNA1/donor DNA with or without prior treatment with the c-Kit ligand, SCF or with the inhibitor, dasatinib. Data are presented as mean ± s.e.m., n = 3; analysis by student's t-test, *$p < 0.05$.

Supplementary Fig. 8

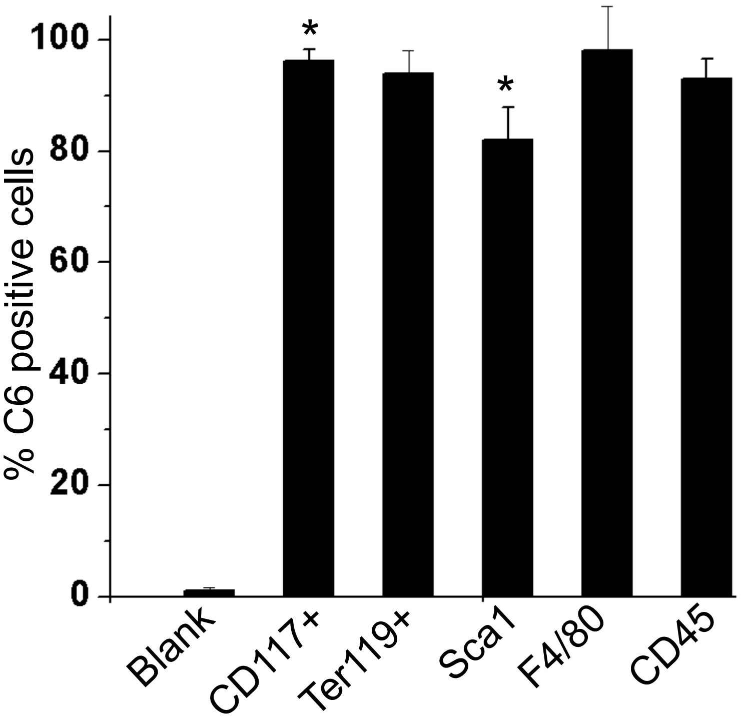

Supplementary Fig. 8. Uptake of PLGA NPs by sub-populations of hematopoietic cells expressing the indicated markers. (Related to Fig. 2.) NPs were formulated to contain the dye Coumarin 6 (C6) so that uptake into the cells could be quantified by flow cytometry. Data are shown as mean ± s.e.m., n = 3; analysis by student's t-test, $*p < 0.05$.

Supplementary Fig. 9

A

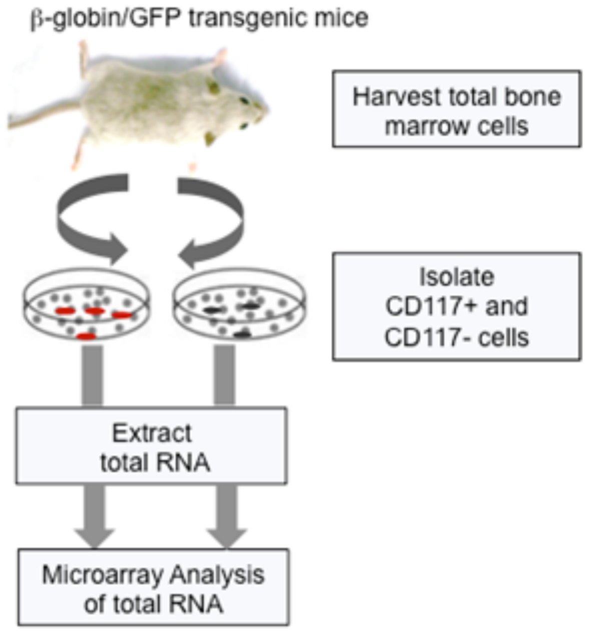

B

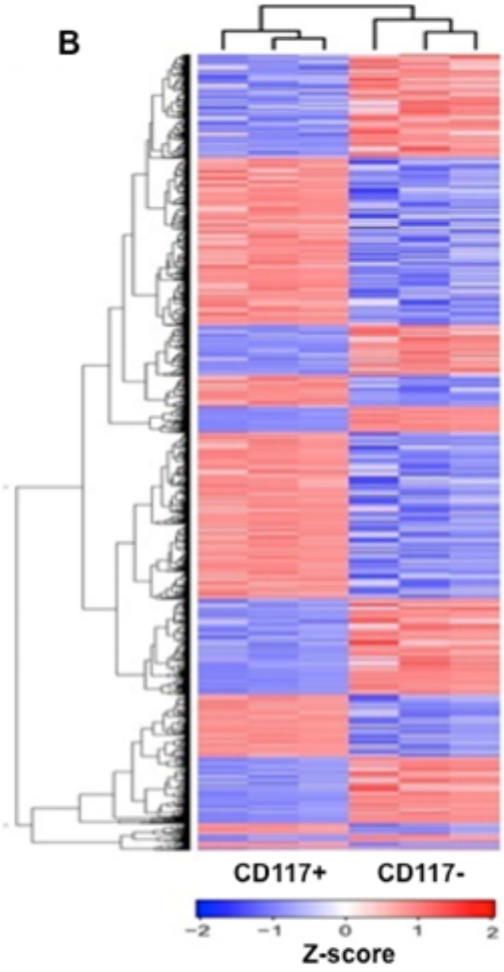

C

Gene	CD117 Negative	CD117 Positive	Fold Change CD117 negative/ CD117 positive	P value
c-Kit	593.98	2368.32	-3.98715	0.0051
VEGF	344.34	1109.97	-3.22341	0.0084
Sca1	208.24	490.86	-2.35711	0.0126
Erdr1	1011.81	2760.26	2.72805	0.0319

Supplementary Fig. 9. Gene expression analysis in CD117+ cells versus CD117- cells. (Related to Fig. 2). (A) Experimental design for the analysis of gene expression profiles of total RNA from CD117+ and CD117- cells isolated from bone marrow of β-globin/GFP transgenic mice (n=3). (B) Gene expression profile comparison of CD117+ versus CD117- cells isolated from bone marrow of β-globin/GFP transgenic mice. All the differentially expressed genes with a false discovery rate less than 0.05 are shown; rows are clustered by Euclidean distance measure. Out of 45282 transcripts, 7890 showed up-regulation in CD117+ enriched cells. (C) Table showing selected genes that were up-regulated in CD117+ enriched cells as compared to CD117- cells with increased expression of transcripts expected to be associated with CD117 including c-Kit, VEGF (vascular endothelial growth factor), Sca1 (stem cell antigen-1), and Erdr1 (erythroid differentiation regulator 1). Data are mean ± s.e.m., n = 3; analysis by student's t-test, $^*p < 0.05$.

Supplementary Fig. 10.

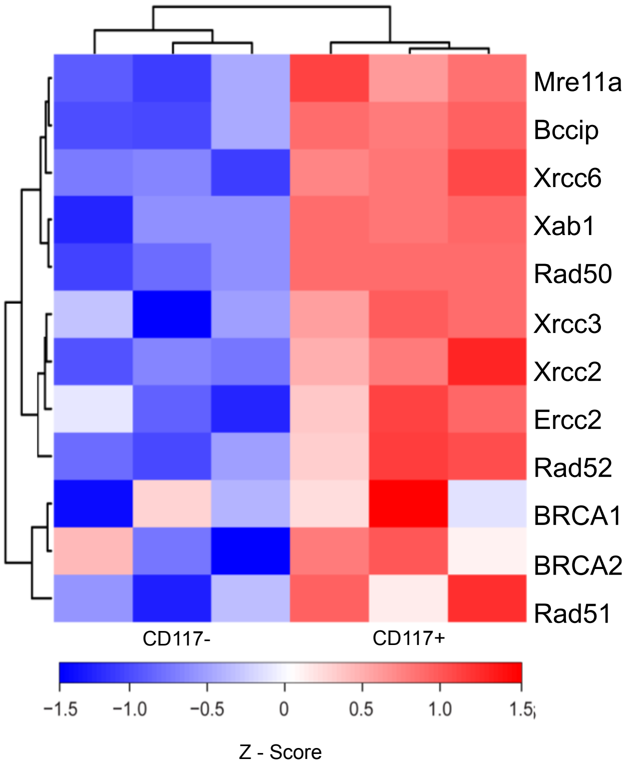

Supplementary Fig. 10. Gene expression analysis in c-Kit+ cells. (Related to Fig. 2). Heat map showing up-regulated genes involved in DNA repair pathways in CD117+ as compared to CD117- cells; rows are clustered by Euclidean distance measure.

Supplementary Fig. 11

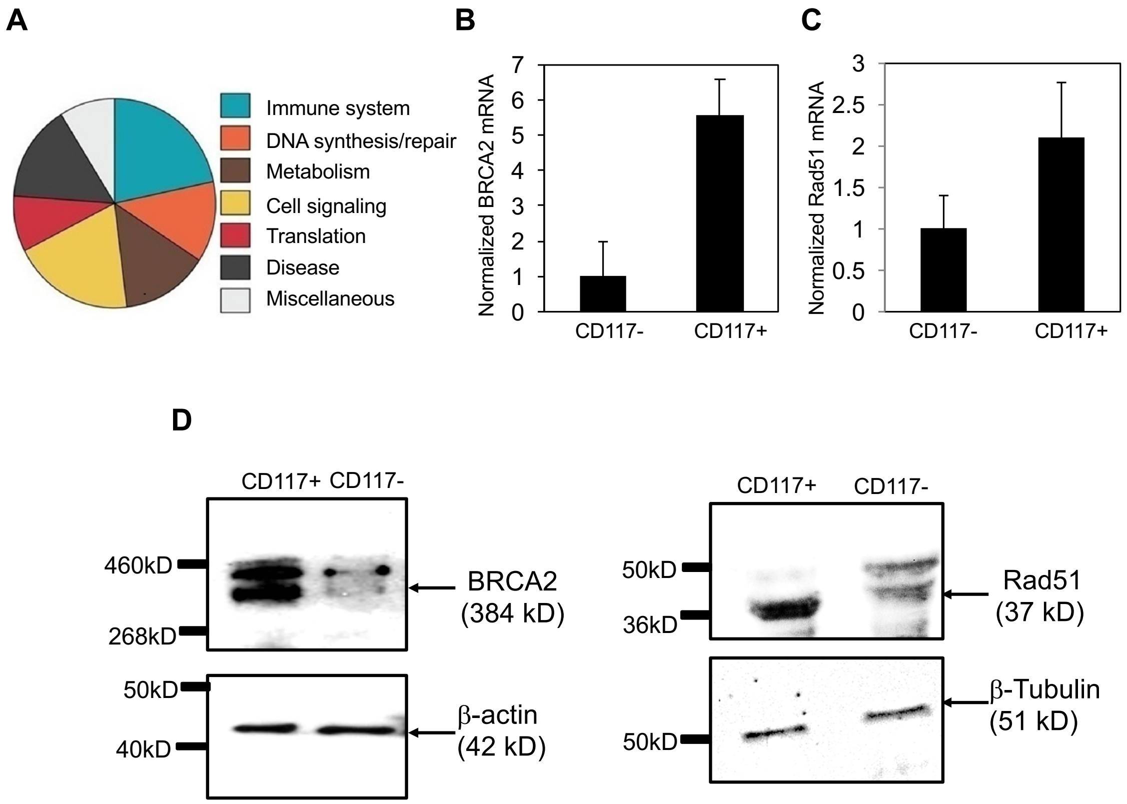

Supplementary Fig. 11. Additional gene expression analyses in CD117+ cells. (Related to Fig. 2.) (A)KEGG pathway analysis of significantly upregulated genes in CD117+ cells. **(**B) and (C) qPCR determination of mRNA expression levels of BRCA2 and Rad51 in CD117- and CD117+ cells. Data are shown as mean ± s.e., n = 3. (D) Western blots showing the levels of BRCA2 protein and Rad51 protein in CD117+ versus CD117- cells. Uncropped images of the corresponding western blot is shown in supplementary figure 23 and supplementary figure 24.

Supplementary Fig. 12

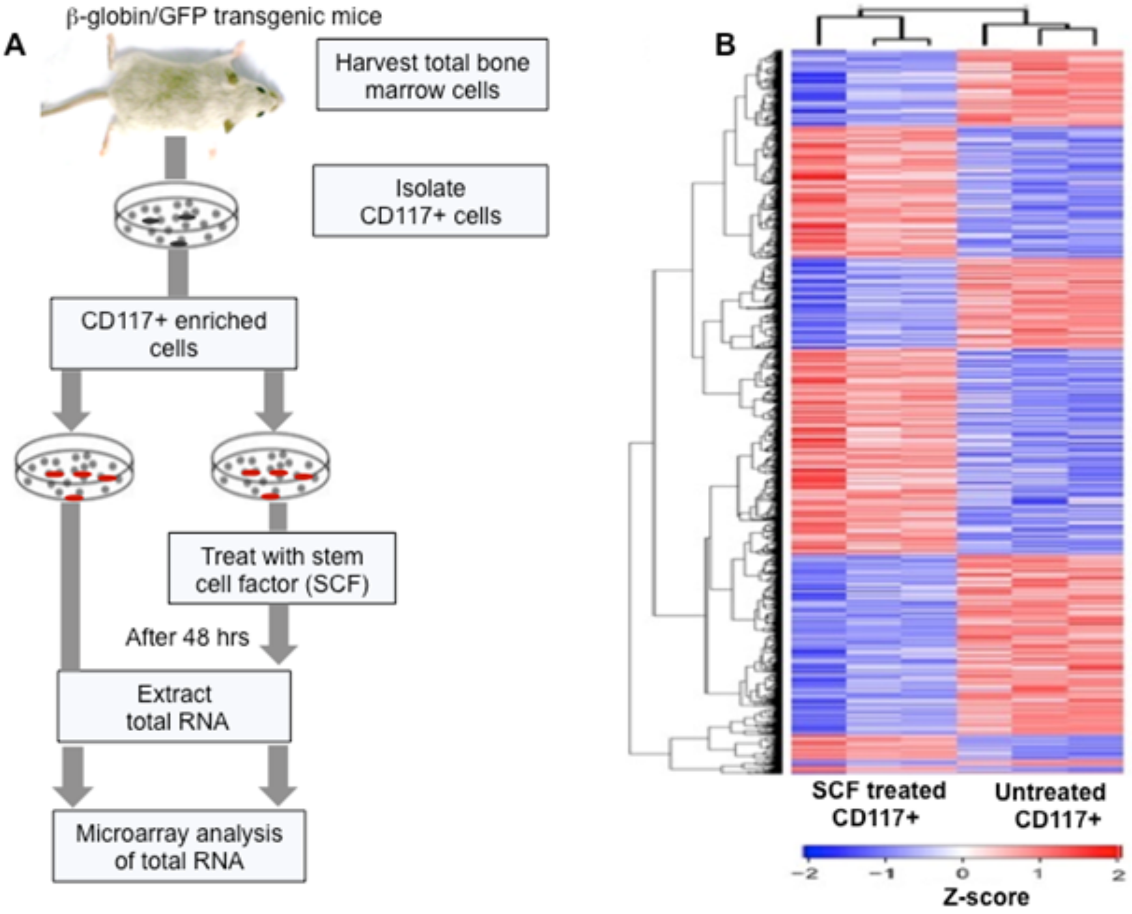

Supplementary Fig. 12. Gene expression analysis in SCF-treated CD117+ cells versus untreated CD117+ cells. (Related to Fig. 2). (A) Experimental design for analysis of gene expression profiles of total RNA isolated from untreated CD117+ cells versus stem cell factor (SCF)-treated CD117+ cells, from bone marrow of β-globin/GFP transgenic mice (n=3). Cells were treated with 3μg/ml of SCF. (B) Gene expression profile analysis of untreated CD117+ cells versus SCF-treated CD117+ isolated from bone marrow of mice. As above, the differentially expressed genes with a false discovery rate less than 0.05 are shown; rows are clustered by Euclidean distance measure.

Supplementary Fig. 13

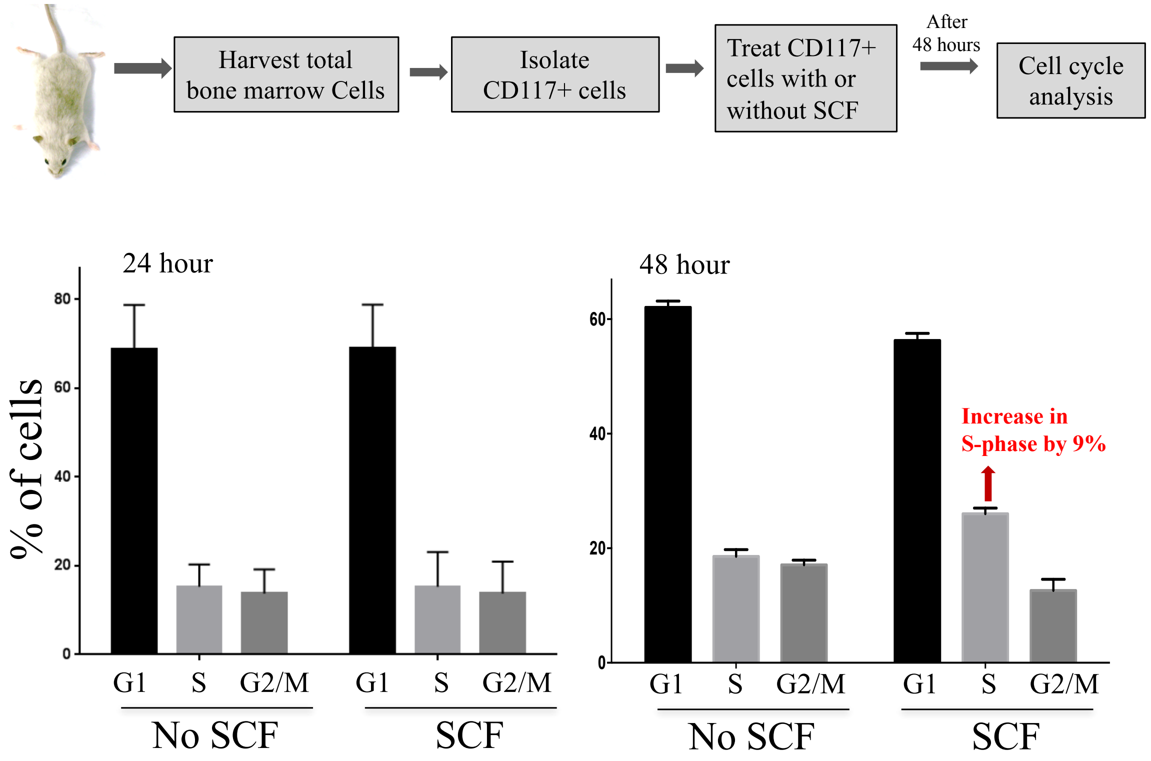

Supplementary Fig. 13. Cell cycle phase analyses. (Related to Fig. 2.) Effect of SCF on cell cycle phase distribution of CD117+ cells at 24 and 48 h after SCF treatment *ex vivo.* Data are mean ± s.e.m., n =3.

Supplementary Fig. 14

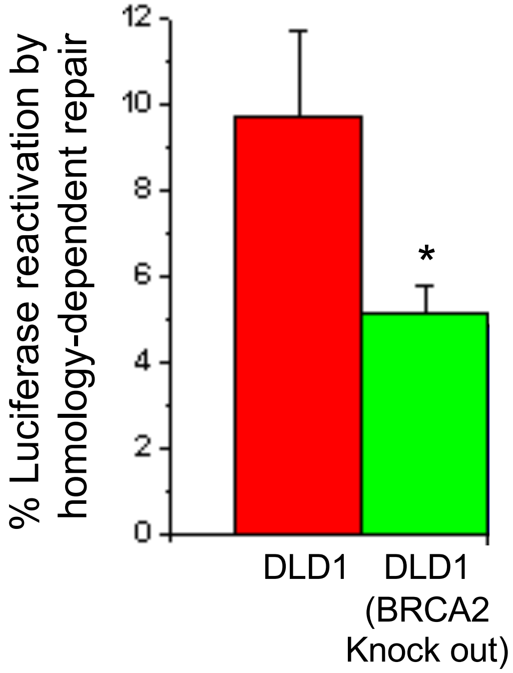

Supplementary Fig. 14. Validation of luciferase reporter assay to quantify homology-dependent repair. (Related to Fig. 3). Results of reporter gene assay for HDR activity in BRCA2-proficient or deficient DLD1 cell lines. Data are mean ± s.e.m., n = 3; analysis by student's t-test, $*p < 0.05$.

Supplementary Fig. 15

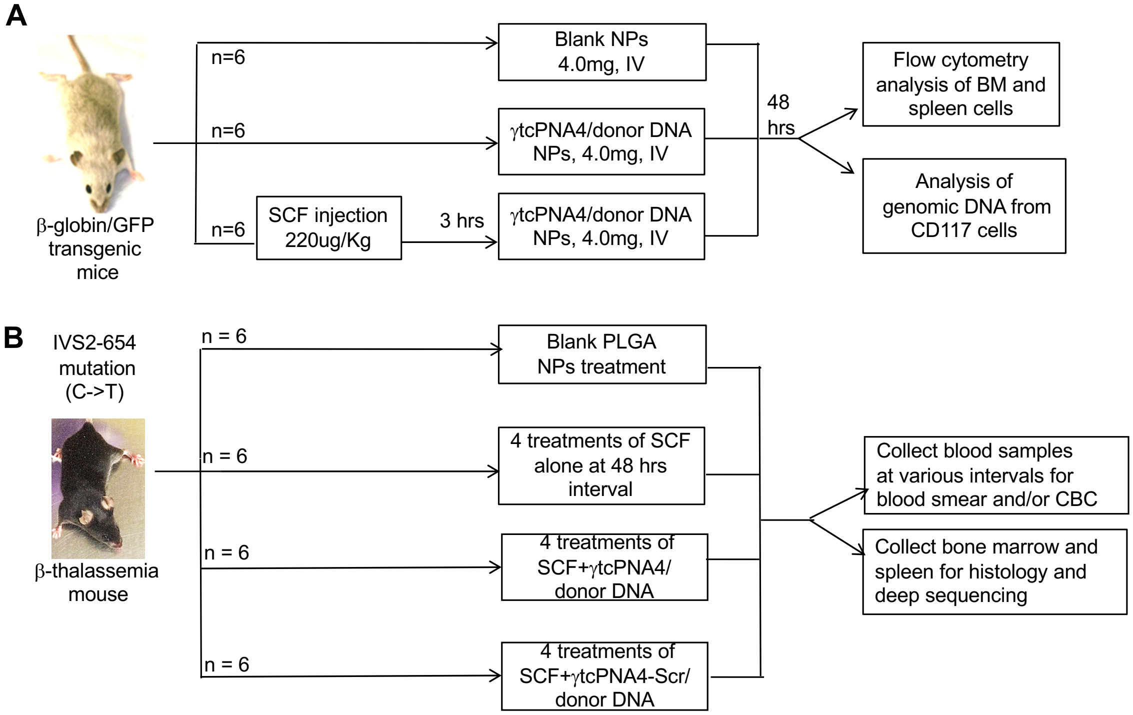

Supplementary Fig. 15. Experimental scheme for PNA NP-mediated *in vivo* gene editing in β-globin/GFP and β-thalassemia mouse models. (Related to Figs. 3, 4, and 5). Work flow to test for PNA-mediated gene editing *in vivo* in (A) β-globin/GFP and (B) the β-thalassemia mouse models.

Supplementary Fig. 16

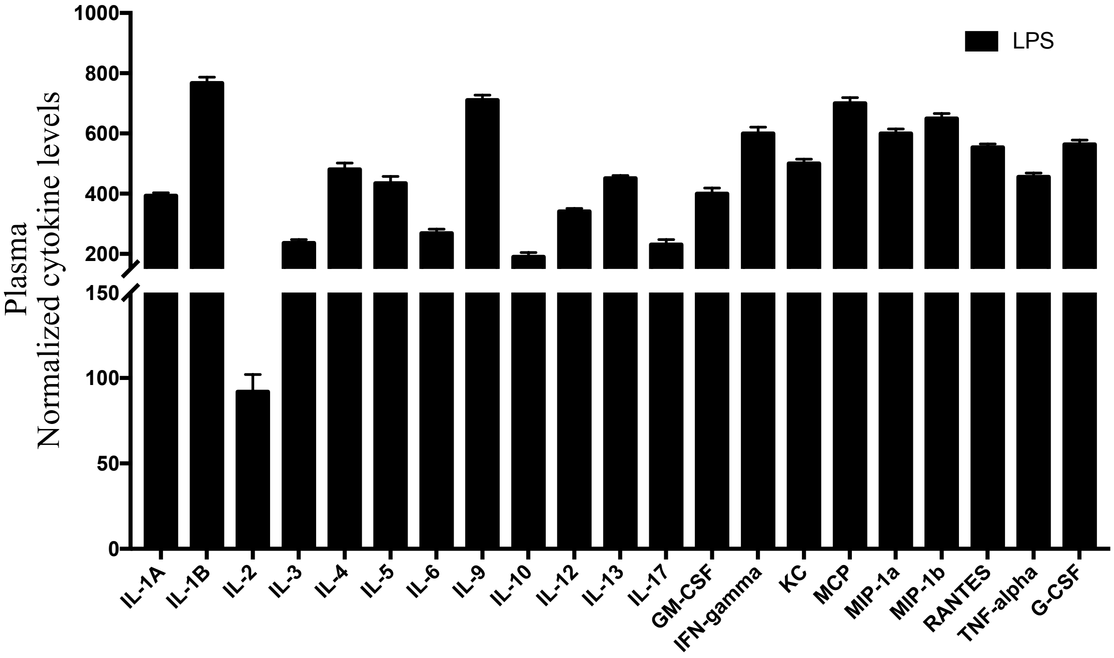

Supplementary Fig. 16 (Related to Fig. 3)**.** Analysis of cytokine levels, as indicated, in blood of lipopolysaccharide (LPS) (200ug/mouse) treated mice at 48 h post treatment. These data provide a positive control for Fig. 3D, but are presented separately because they would be off scale in Fig. 3D. Data are mean ± s.e.m., n = 3.

Supplementary Fig. 17

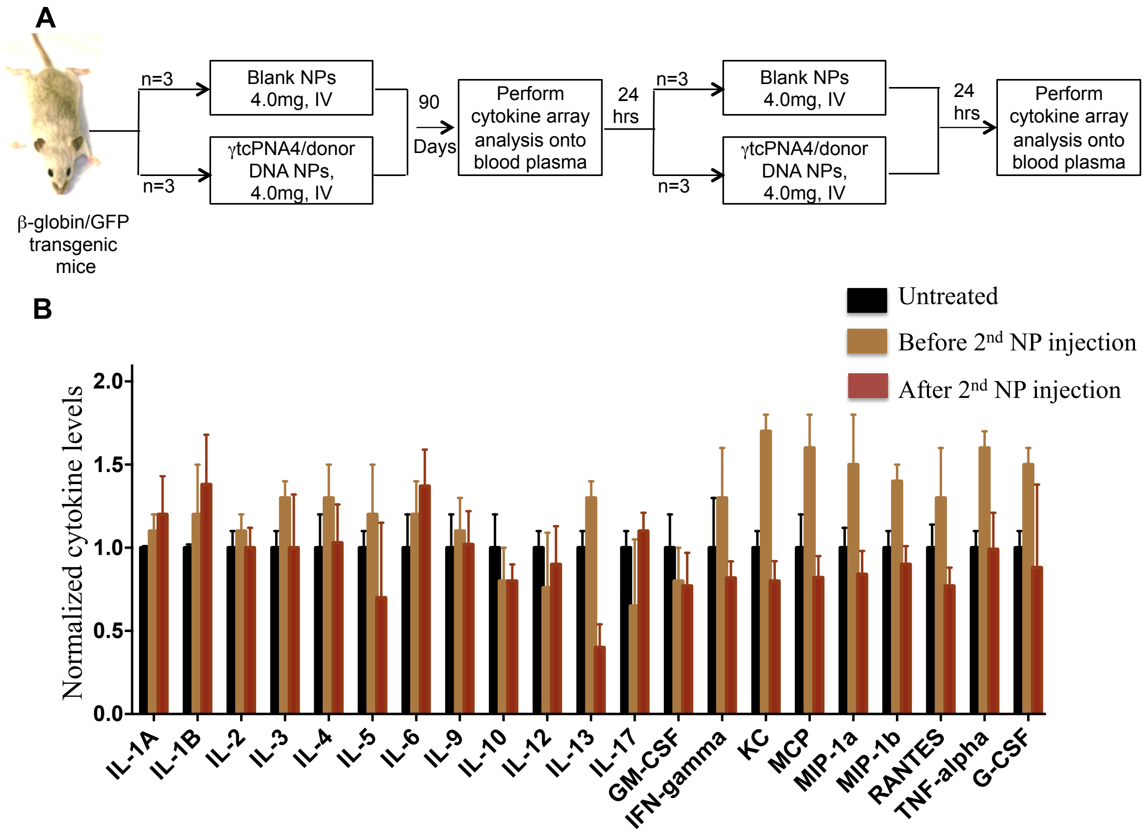

Supplementary Fig. 17. Analysis of immune and inflammatory responses upon repeat treatment of mice with PNA-containing nanoparticles. (Related to Figs. 3, 4, and 5). (A) Schematic showing long-term immunogenicity experiment performed using β-globin/GFP transgenic mice. Mice were treated with NPs as indicated on day 1, and then again 90 days later. Blood samples were obtained on the day before and on the day after the second treatment for analysis of cytokine levels in plasma as indicated in (B). Data are mean ± s.e.m., n = 3.

Supplementary Fig. 18

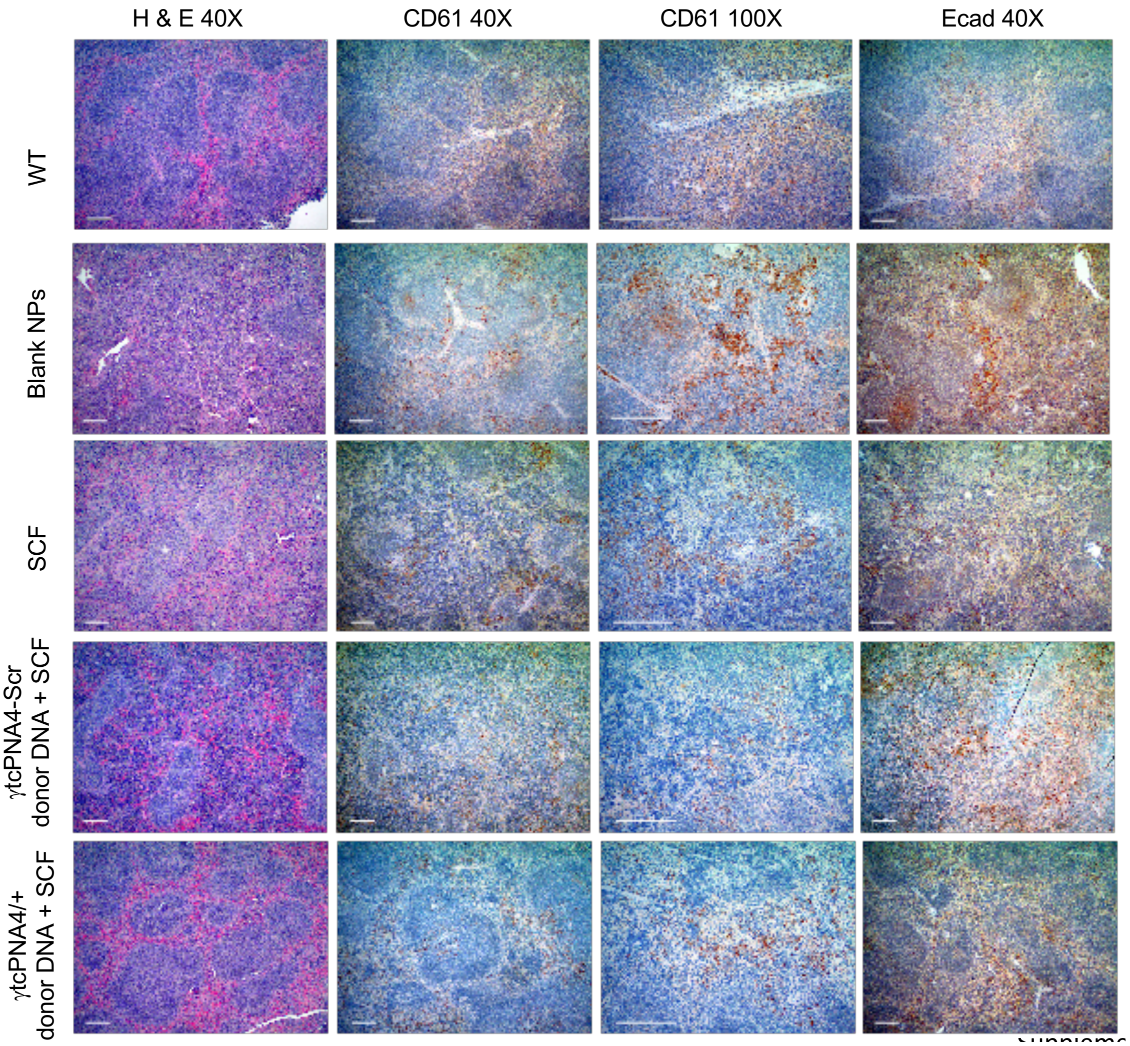

Supplementary Fig. 18. Histopathologic analysis of spleen sections from wild-type mice and from thalassemic mice obtained 36 days after treatment with blank NPs, SCF alone, SCF plus scrambled γtcPNA4-Scr/donor DNA NPs, or SCF plus γtcPNA4/donor DNA NPs. (Related to Fig. 4). Hematoxylin and eosin stain (H&E), 40x magnification; CD61, 40x and 100x magnification; E-cadherin (Ecad), 40x magnification, as indicated. Scale bar, 10.0 µm.

Supplementary Fig. 19

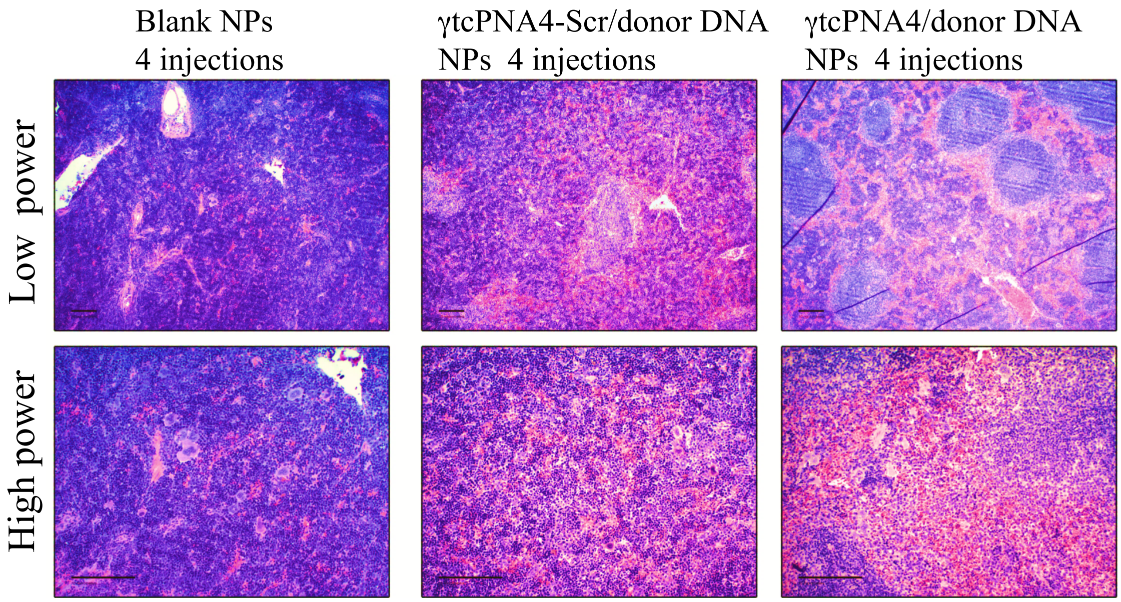

Supplementary Fig. 19. Histopathologic analysis of spleen sections from from thalassemic mice obtained 75 days after treatment with blank NPs, SCF plus scrambled γtcPNA4-Scr/donor DNA NPs, or SCF plus γtcPNA4/donor DNA NPs. (Related to Fig. 4). Hematoxylin and eosin stain (H&E), 40x magnification and 100x magnification; as indicated. Scale bar, 10.0 µm.

Supplementary Fig. 20

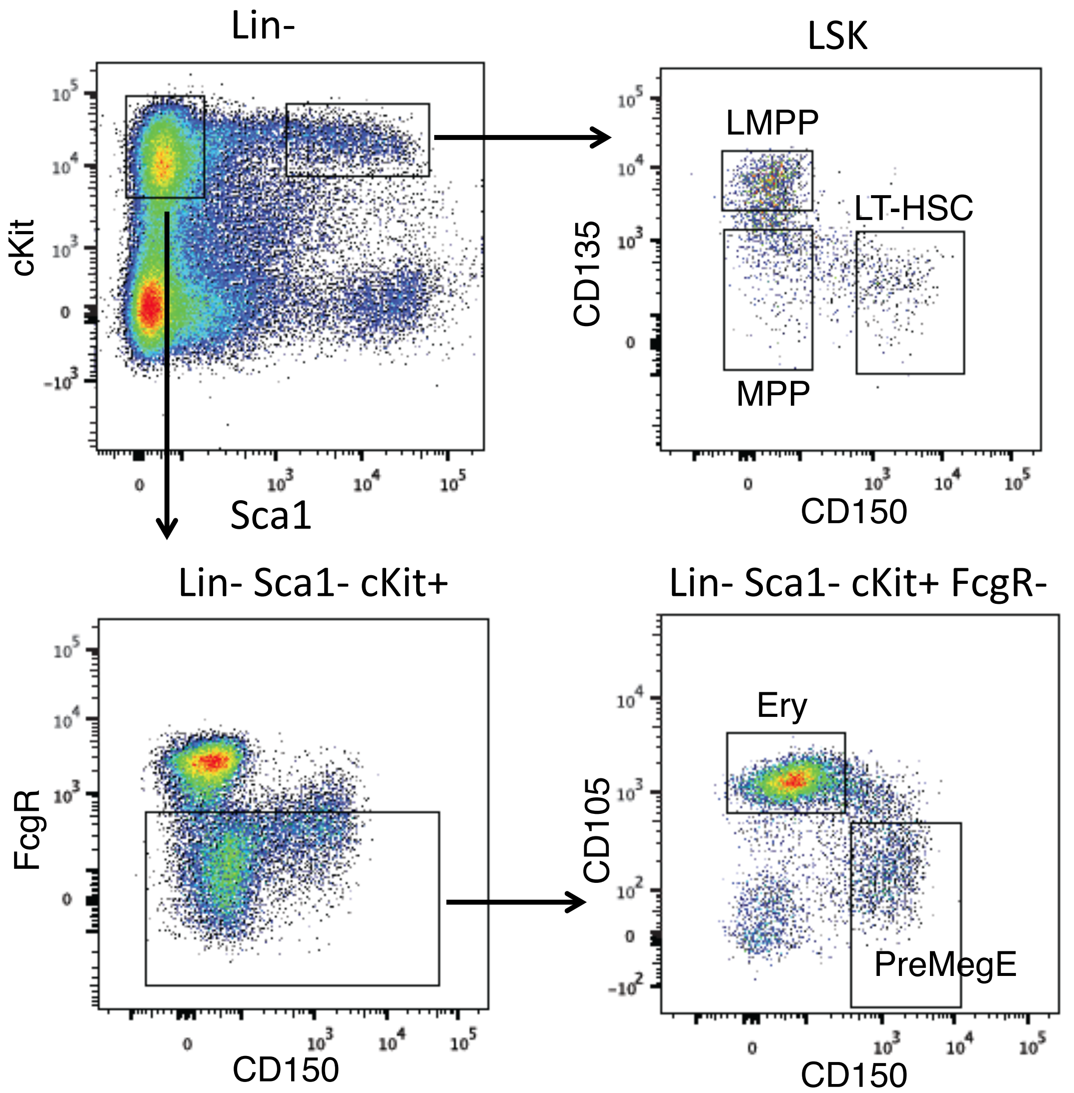

Supplementary Fig. 20. FACS sorting scheme. (Related to Fig. 5). FACS sorting scheme for isolation and analysis of hematopoietic stem and progenitor cells from the bone marrow of treated β-thalassemic mice.

Supplementary Fig. 21

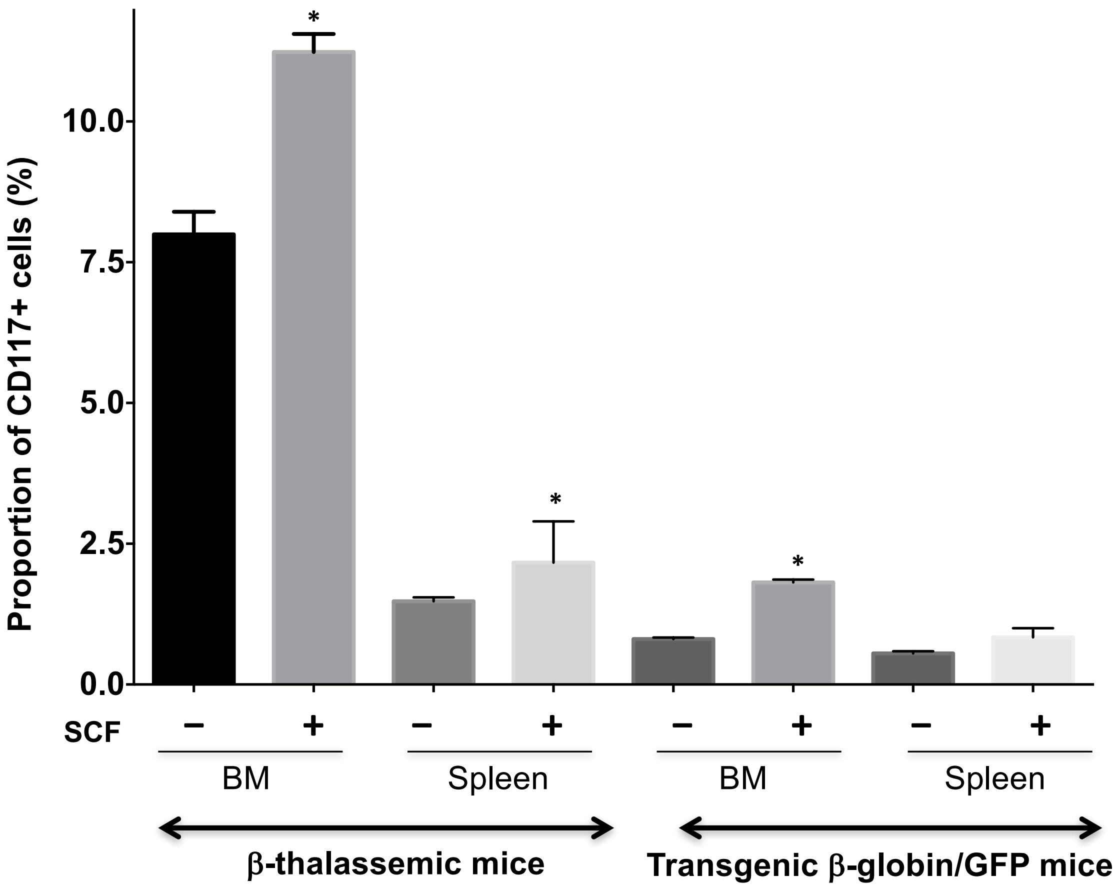

Supplementary Fig. 21. (Related to Figs. 3, 4, and 5.) Percentage of CD117 cells out of total cells derived from bone marrow and spleen of β-thalassemic and β-globin/GFP transgenic mice after *in vivo* treatment with and without SCF (ip). Data are mean ± s.e.m., n = 3; analysis by student's t-test, *$p < 0.05$.

Supplementary Fig. 22

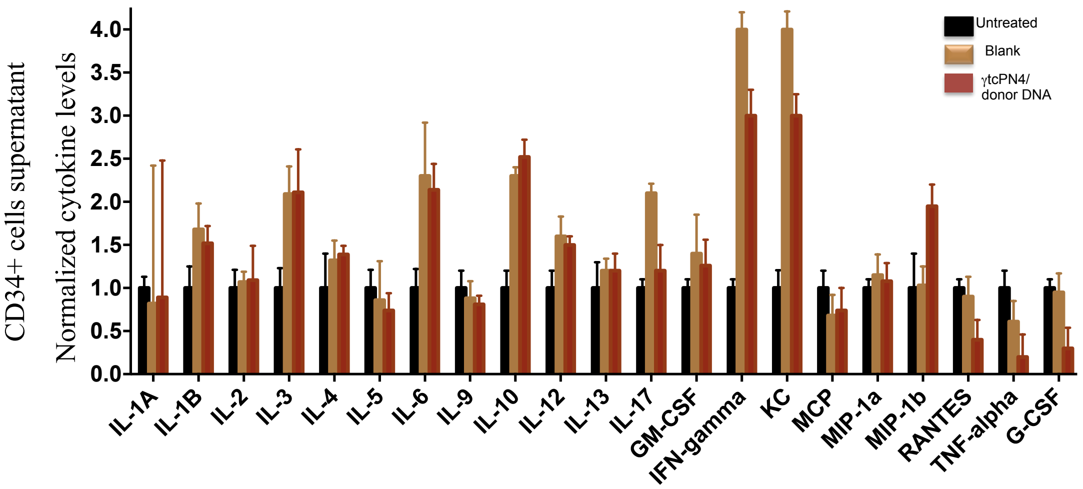

Supplementary Fig. 22. (Related to Fig. 5). Cytokine array analysis performed on supernatant collected from human CD34+ cells 24 h after the indicated treatments. Data are mean ± s.e.m., n = 3.

Supplementary Fig. 23.

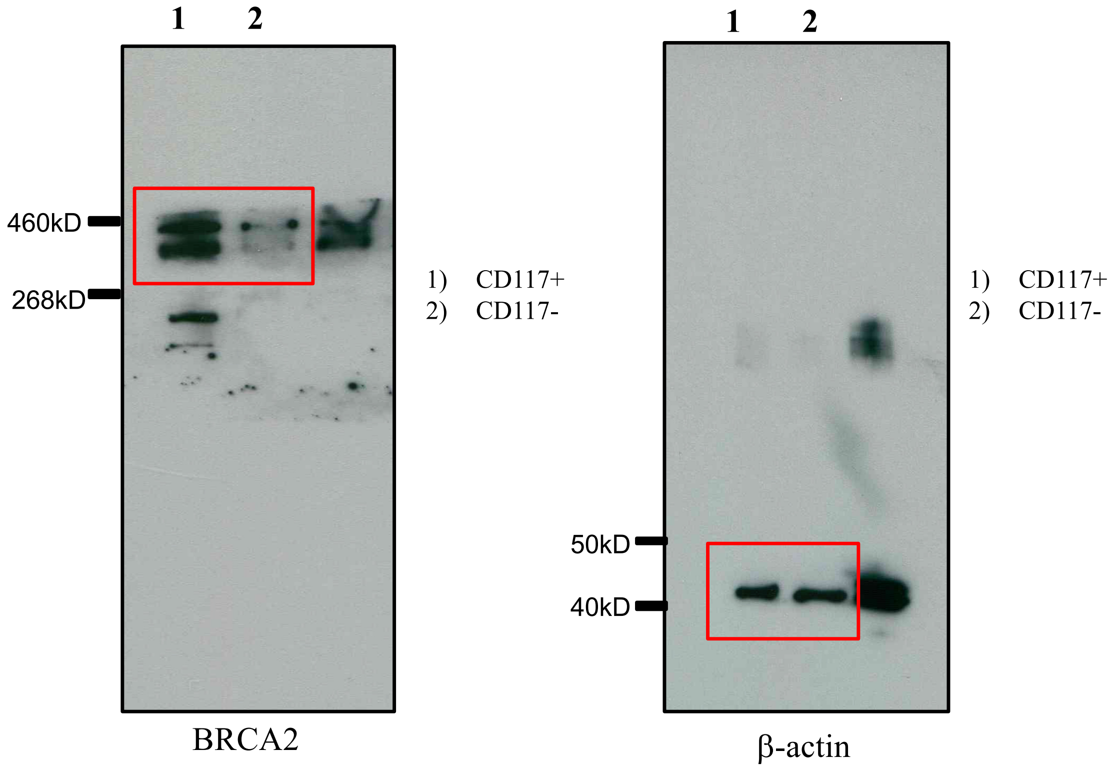

Supplementary Fig. 23. Uncropped western blots for analysis of BRCA2 and β-actin expression (Related to Supplementary Fig. 11D).

Supplementary Fig. 24.

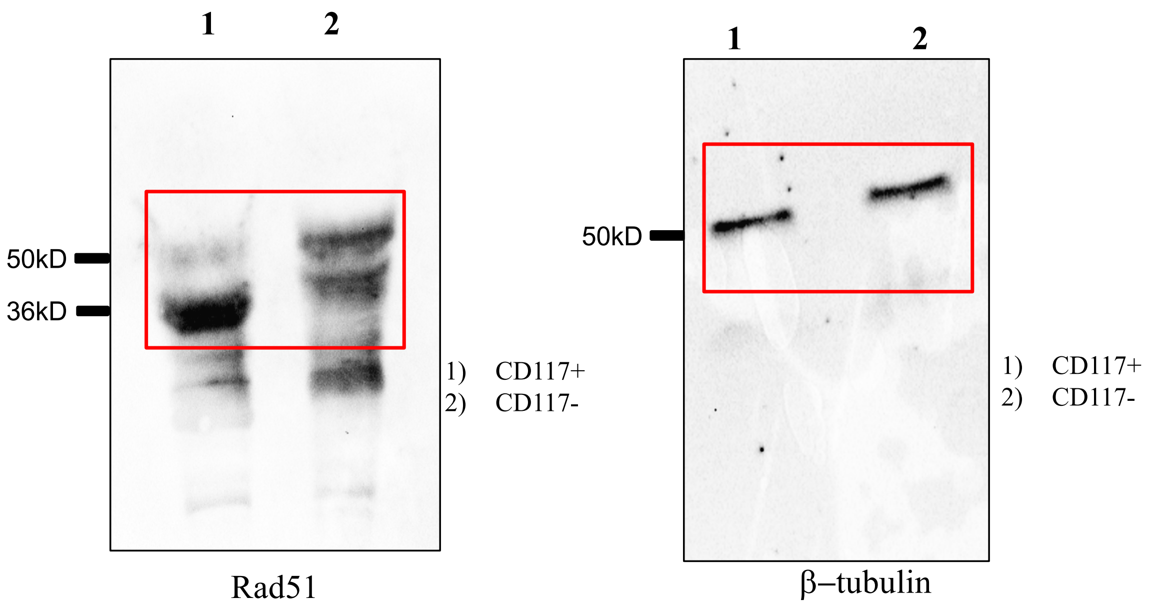

Supplementary Fig. 24. Uncropped western blots of Rad51 and β-tubulin expression (Related to Supplementary Fig. 11D).

tcPNAs Probes	Theoretical molecular weight	Observed molecular weight
tcPNA1	8911.07	8913.21
tcPNA2	7578.88	7579.32
tcPNA3	7248.52	7250.34
γtcPNA4	9980.71	9982.98

Supplementary Table 1. MALDI-TOF analyses of synthesized tcPNAs. (Related to Fig. 1.)
